# Diptera of Canada

**DOI:** 10.3897/zookeys.819.27625

**Published:** 2019-01-24

**Authors:** Jade Savage, Art Borkent, Fenja Brodo, Jeffrey M. Cumming, Gregory Curler, Douglas C. Currie, Jeremy R. deWaard, Joel F. Gibson, Martin Hauser, Louis Laplante, Owen Lonsdale, Stephen A. Marshall, James E. O’Hara, Bradley J. Sinclair, Jeffrey H. Skevington

**Affiliations:** 1 Bishop’s University, Sherbrooke, Quebec, Canada Bishop's University Sherbrooke Canada; 2 Agriculture and Agri-Food Canada, Canadian National Collection of Insects, Arachnids and Nematodes, Ottawa, Ontario, Canada Royal British Columbia Museum Salmon Arm Canada; 3 Royal British Columbia Museum, Victoria, British Columbia, Canada Canadian Museum of Nature Ottawa Canada; 4 Mississippi Entomological Museum, Mississippi State University, Starksville, Mississippi, USA Agriculture and Agri-Food Canada Ottawa Canada; 5 Royal Ontario Museum, Toronto, Ontario, Canada Mississippi State University Starksville United States of America; 6 Centre for Biodiversity Genomics, University of Guelph, Guelph, Ontario, Canada Royal Ontario Museum Toronto Canada; 7 California Department of Food and Agriculture, Sacramento, California, USA University of Guelph Guelph Canada; 8 Unaffiliated, Montreal, Quebec, Canada Royal British Columbia Museum Victoria Canada; 9 University of Guelph, Guelph, Ontario, Canada California Department of Food and Agriculture Sacramento United States of America; 10 Canadian Food Inspection Agency, Ottawa, Ontario, Canada Unaffiliated Montreal Canada; 11 Canadian Museum of Nature, Ottawa, Ontario, Canada Canadian Food Inspection Agency Ottawa Canada

**Keywords:** biodiversity assessment, Biota of Canada, Diptera, flies, systematics

## Abstract

The Canadian Diptera fauna is updated. Numbers of species currently known from Canada, total Barcode Index Numbers (BINs), and estimated numbers of undescribed or unrecorded species are provided for each family. An overview of recent changes in the systematics and Canadian faunistics of major groups is provided as well as some general information on biology and life history. A total of 116 families and 9620 described species of Canadian Diptera are reported, representing more than a 36% increase in species numbers since the last comparable assessment by JF McAlpine et al. (1979). Almost 30,000 BINs have so far been obtained from flies in Canada. Estimates of additional number of species remaining to be documented in the country range from 5200 to 20,400.

*This paper is dedicated to the memory of Terry A Wheeler, an exceptional Canadian dipterist and long-time contributor to the Biological Survey of Canada, who passed away in the early stages of this project*.

The world fauna of Diptera counts almost 160,000 named species (Borkent et al. 2018) divided into approximately 160 extant families (Pape and Thompson 2013). Flies dominate the Canadian insect fauna in numbers of named species and, in many habitats, in overall abundance. That dominance becomes especially apparent in the northern parts of the country where dipterans form a ubiquitous feature of the summer landscape.

Diptera occur in almost all freshwater and terrestrial habitats where they display an impressive range of life histories and feeding habits. From parasites to leafminers, predators and filter feeders (to mention only a few), flies have diversified to exploit almost all organic substrates for their development (see Courtney et al. (2017) and Marshall (2012) for detailed overviews). Canada holds approximately 20% of the world’s freshwater reserves so, unsurprisingly, families with aquatic stages are very well represented in the country. In the present survey, the Chironomidae (non-biting midges), whose immature stages are primarily aquatic, account for the most named species (798) in a single family (Table 1).

The diversity of flies in Canada was last reviewed by JF McAlpine et al. (1979) as part of a broader treatment of the terrestrial arthropods (Danks 1979). Subsequently, the three volumes of the *Manual of Nearctic Diptera* (JF McAlpine et al. 1981, 1987, JF McAlpine and Wood 1989) have been major catalysts for dipterological research in Canada and the USA. A detailed overview of these contributions, and the people who made them possible, was provided in Cumming et al. (2011). The identification keys to genus found in Volumes 1 and 2 (JF McAlpine et al. 1981, 1987) paved the way for future taxonomic work on the Nearctic fauna, and for many families they remain the best identification resource. While recent catalogues are now available for a number of Nearctic Diptera families, e.g., Dolichopodidae (Pollet et al. 2004) and Tachinidae (O’Hara and Wood 2004), no comprehensive catalogue has been published for the Canadian fauna of the whole order since Stone et al. (1965). The global online database, Systema Dipterorum (Pape and Thompson 2013), provides extensive information about Diptera names and literature; it is especially useful for resolving issues related to precedence and validity of names during taxonomic revisions.

As with many arthropod groups, the development of DNA-based identification and phylogenetic tools has had a strong impact on Diptera systematics. DNA barcoding using a part of the cytochrome c oxidase 1 (COI) gene (Hebert et al. 2003) has been applied to members of nearly all Diptera families found in Canada, and more specimens of flies have been DNA barcoded than of any other order in the country – 1.14M specimens as of June 2018 in the Barcode of Life Data System (BOLD; www.boldsystems.org). DNA barcoding and the Barcode Index Number (BIN) system (Ratnasingham and Hebert 2013) usually provide good estimates of species limits in taxa with good coverage, e.g., Canadian Muscidae (Renaud et al. 2012b, Hebert et al. 2016) and Simuliidae (Rivera and Currie 2009). However, gaps and errors in existing barcode libraries in some freshwater taxa (Curry et al. 2018), as well as poor correspondence between COI DNA barcodes and morphology for at least one genus found in Canada (*Protocalliphora* Hough; Whitworth et al. 2007) warrant caution when using BINs alone as estimates of true Diptera species diversity. In any case, further investigations will be required to explore the great discrepancies between named species and BINs for some families such as the Cecidomyiidae (243 vs 11,396) or the Sciaridae (129 vs 2863) (Table 1), and to determine the relative contributions of gaps in taxonomic knowledge and discordance with the DNA barcoding and/or BIN approach.

The *Manual of Nearctic Diptera*, especially Volume 3 (McAlpine and Wood 1989), also had a major impact on the field of Diptera phylogenetics. The hypotheses of family-level relationships and the proposed classification presented have served as a basis for future updates (Yeates and Wiegmann 2012) and have since been tested repeatedly using new sources of data and continuously evolving quantitative methods (see Wiegmann and Yeates (2017) for review). It is notable that while the last three decades have generated an impressive body of literature on Diptera phylogenetics, a lack of consensus still remains in many parts of the Diptera phylogeny (Borkent 2018). Consequently, the family concepts used in the present work follow Pape et al. (2011) but the classification reflects a consensus of opinions of co-authors and collaborators who have contributed data to this paper.

When compared to the data provided in JF McAlpine et al. (1979), the results of the present work (Table 1) show an increase from 101 to 116 families (excluding the unranked *Iteaphila* group formerly placed in Empididae). While the Oreoleptidae and the Richardiidae represent new records for the country, most of the additional families represent reclassification of taxa formerly combined with other families (see text below and Table 1 for details). The numbers of recorded and named species have also increased since 1979 for most families, with the exception of those that were split (e.g., Tipulidae and Empididae) or those in which numerous synonymies were uncovered (e.g., Bibionidae). Especially worth noting are the Sphaeroceridae and Anthomyzidae, with five- and nine-fold increases in species numbers since 1979, respectively. In each of these cases, the impressive increase in species numbers can be attributed to decades-long dedication to biosystematics study of particular families by individuals and institutions (S Marshall and colleagues at the University of Guelph, Ontario, for the Sphaeroceridae and K Barber at the Great Lakes Forestry Centre in Sault Ste. Marie, Ontario, for the Anthomyzidae).

JF McAlpine et al. (1979) compiled 7056 species of Diptera in Canada (mistakenly reported as 7058 in table 42) and estimated that an approximately equivalent number remained to be discovered. The 9620 species reported here represents a 36% increase since 1979. Significant advances have been made over the last four decades but some major gaps remain. While few families are known well enough to claim full coverage in Canada, the bulk of undescribed or unrecorded Canadian Diptera diversity is in the nematocerous families, especially those with diminutive and/or delicate adults such as the Chironomidae, Ceratopogonidae, Cecidomyiidae and Mycetophilidae, all of which are in great need of taxonomic attention (Table 1).

**Table 1.** Census of Diptera in Canada.

Taxon^1^	No. species reported in McAlpine et al. (1979)	No. species^2^ currently known from Canada	No. BINs^3^ available for Canadian species	Est. no. undescribed or unrecorded species in Canada	General distribution by ecozone^3A^	Information sources
**Nematocerous Diptera**						
**Infraorder Tipulomorpha**						
**Superfamily Tipuloidea**						
Tipulidae	520^4^	216	190	30	all ecozones	Oosterbroek 2018
Cylindrotomidae	? ^4^	7	6	0	Pacific Maritime, Boreal Plains, Boreal Shield, Newfoundland Boreal, Mixedwood Plains, Atlantic Maritime	Oosterbroek 2018
Limoniidae	? ^4^	354	345	186	all ecozones	Oosterbroek 2018
Pediciidae	? ^4^	56	52	0	all but Arctic	Oosterbroek 2018
**Superfamily unassigned**						
Trichoceridae	20	21	34	10	all ecozones	Pratt 1992
**Infraorder unassigned**						
Deuterophlebiidae	1	3	1	1	Boreal Cordillera, Montane Cordillera	
Nymphomyiidae	2	1	1	0	Boreal Shield, Atlantic Maritime, Mixedwood Plains	Courtney 1994
**Infraorder Psychodomorpha**						
Blephariceridae	11	7	4	3	all but Prairies	
Psychodidae	30	34	114	10−50	all ecozones	Quate 1955, Young and Perkins 1984
Tanyderidae	2	2	0	1	Montane Cordillera, Atlantic Maritime	
**Infraorder Ptychopteromorpha**						
Ptychopteridae	7	8	5	8	all but Arctic	Fasbender and Courtney 2017
**Infraorder Culicomorpha**						
**Superfamily Chironomoidea**						
Chironomidae	480	798	4266	1000	all ecozones	Ashe and O’Connor 2009, 2012
**Superfamily Simulioidea**						
Ceratopogonidae	180	263	1341	300	all ecozones	Borkent and Grogan 2009, Borkent 2016
Thaumaleidae	3	13	6	2−5	Pacific Maritime, Boreal Shield, Newfoundland Boreal, Montane Cordillera, Mixedwood Plains, Atlantic Maritime	Pivar et al. 2018
Simuliidae	110	164	153	20	all ecozones	Adler and Crosskey 2018
**Superfamily Culicoidea**						
Dixidae	21	34	23	10	all ecozones	
Corethrellidae	? ^5^	1	1	0	Mixedwood Plains	Borkent 2008, 2014
Chaoboridae	9^5^	11	21	0	all ecozones	Borkent 1979, 1981, 2014
Culicidae	74	82	75	3	all ecozones	Thielman and Hunter 2007, Jackson et al. 2013, Giordano et al. 2015
**Infraorder unassigned**						
Axymyiidae	1	1	1	2	Mixedwood Plains	
**Infraorder Bibionomorpha s. lat.**						
Anisopodidae	5	5	15	2−5	all but Arctic	
**Superfamily Scatopsoidea**						
Scatopsidae	30	30	48	15−20	all ecozones	
Canthyloscelidae	1^6^	1	1	0	all but Arctic	
**Infraorder Bibionomorpha s. str.**						
Pachyneuridae	1	1	1	0	Pacific Maritime	
Bibionidae	40^7^	26	29	2−3	all ecozones	
Hesperinidae	?^7^	1	1	0	all Boreal ecozones	Papp 2010
**Superfamily Sciaroidea**						
Ditomyiidae	?^8^	3	9	5−10	all but Arctic	Munroe 1974
Bolitophilidae	? ^8^	16	23	5	all ecozones	Bechev and Chandler 2011
Keroplatidae	? ^8^	28	95	4	all ecozones	Evenhuis 2006
Mycetophilidae	350^8^	489	1199	500	all ecozones	
Cecidomyiidae	100	243	11,396	1000−16,000	all ecozones	Gagné and Jaschhof 2017
Diadocidiidae	? ^8^	2	8	5	all but Arctic	Bechev and Chandler 2011
Sciaridae	30	129	2863	100−200	all ecozones	Mohrig et al. 2013
**Suborder Brachycera**						
**Infraorder Xylophagomorpha**						
**Superfamily Xylophagoidea**						
Xylophagidae	15	14	16	1−2	all but Arctic, Taiga Shield and Taiga Plains	Woodley 2011c
**Infraorder Tabanomorpha**						
**Superfamily Rhagionoidea**						
Rhagionidae	35^9^	48	57	10−15	all ecozones	Kerr 2010
Bolbomyiidae	? ^9^	3	5	2	Pacific Maritime, Mixedwood Plains	Kerr 2010
**Superfamily Tabanoidea**						
Pelecorhynchidae	4	5	1	1	Montane Cordillera, Mixedwood Plains	
Oreoleptidae	0	1	2	0−1	Montane Cordillera, Boreal Cordillera	Zloty et al. 2005
Athericidae	3	2	3	0	all but Arctic and Prairies ecozones	
Tabanidae	132	142	90	0	All but Arctic	
**Infraorder Stratiomyomorpha**						
**Superfamily Stratiomyoidea**						
Xylomyidae	4	7	8	0	Pacific Maritime, Western Interior Basin, Boreal Shield, Mixedwood Plains, Atlantic Maritime	Woodley 2011a,b
Stratiomyidae	84	114	71	5−10	all but Arctic	Woodley 2001, 2011a
**Infraorder unassigned**						
Acroceridae	20	20	14	5	all but Arctic	
Nemestrinidae	2	2	0	0	Western Interior Basin	
**Infraorder Asilomorpha**						
**Superfamily Asiloidea**						
Bombyliidae	70^10^	105	86	44	all ecozones	
Mythicomyiidae	? ^10^	1	2	5	all but Arctic, Taiga Shield, and Taiga Plains	Evenhuis 2002
Hilarimorphidae	7	7	1	1	all but Arctic	Webb 1974, 1975
Asilidae	125	222	141	5−10	all but Arctic	
Mydidae	2	2	1	0	Western Interior Basin, Mixedwood Plains	
Apioceridae	1	1	0	0	Western Interior Basin	
Scenopinidae	8	10	3	1−5	all but Arctic	
Therevidae	30	50	28	0	all ecozones	Webb et al. 2013
**Infraorder Eremoneura**						
**Superfamily Empidoidea**						
*Iteaphila* group	?^11^	17	23	9	all ecozones	
Oreogetonidae	? ^11^	7	8	1−2	all but Arctic	
Empididae	300^11,12^	251	497	200	all ecozones	
Brachystomatidae	? ^11^	11	11	2	all ecozones	
Hybotidae	? ^11^	155	353	200	all ecozones	
Dolichopodidae s.l.	500^12^	508	657	200	all ecozones	Pollet et al. 2004
**Lower Cyclorrhapha**						
Lonchopteridae	4	7	6	0−1	all ecozones	Klymko and Marshall 2008
**Superfamily Platypezoidea**						
Platypezidae	21	39	46	12	all ecozones	
**Superfamily Phoroidea**						
Phoridae	127	135	110	300	all ecozones	
**Superfamily Syrphidoidea**						
Syrphidae	500	539	359	34	all ecozones	
Pipunculidae	45	85	170	170	all ecozones	
**Schizophora: Acalytratae**						
**Superfamily Diopsidoidea**						
Diopsidae	1	2	2	0	Mixedwood Plains	Feijen 1989
Psilidae	25	27	28	3	all but Arctic	
Strongylophthalmyiidae	1	2	2	0	Pacific Maritime, Prairies, Boreal Shield, Mixedwood Plains, Atlantic Maritime	Barber 2006
Tanypezidae	1	1	1	0	Boreal Shield, Mixedwood Plains	Lonsdale 2013
**Superfamily Neroidea**						
Micropezidae	16	16	11	0	all ecozones	Merritt and Peterson 1976
**Superfamily Sciomyzoidea**						
Sciomyzidae	85	120	143	15	all ecozones	Knutson et al. 1986
Sepsidae	17	19	27	5−10	all ecozones	Ozerov 2005
Conopidae	30	42	34	2−5	all but arctic ecozones	
Coelopidae	4	4	3	0	Arctic, Pacific Maritime, Taiga Shield, Boreal Shield, Atlantic Maritime	Vockeroth 1965, Mathis and McAlpine 2011
Dryomyzidae	7^13^	8	8	1−5	all but Arctic	Mathis and Sueyoshi 2011
Helcomyzidae	? ^13^	1	0	0	Pacific Maritime	
Heterocheilidae	? ^13^	1	0	0	Pacific Maritime	
**Superfamily Lauxanoidea**						
Lauxaniidae	64	78	90	10	all ecozones	
Chamaemyiidae	30	35	94	10	all ecozones	
**Superfamily Tephritidoidea**						
Tephritidae	40	122	82	21	all but Arctic	Foote et al. 1994
Platystomatidae	10	10	7	5	all but Arctic	
Ulidiidae	35^14^	35	29	20	all but Arctic	
Lonchaeidae	97	99	78	13	all but Arctic	
Pyrgotidae	3	3	1	0	Pacific Maritime, Prairies, Mixedwood Plains	
Richardiidae	0	1	0	0	Mixedwood Plains	
Pallopteridae	9	9	7	9	all but Arctic	
Piophilidae	31	31	35	4	all ecozones	Rochefort and Wheeler 2015
**Superfamily Opomyzoidea**						
Agromyzidae	305	450	772	76	all ecozones	
Clusiidae	6	22	22	0	all but Arctic	Caloren and Marshall 1998, Lonsdale and Marshall 2007
Asteiidae	5	5	8	3	all Boreal and Maritime ecozones, Montane Cordillera, Prairies, Mixedwood Plains	
Anthomyzidae	4	37	33	3	all but Arctic	Roháček and Barber 2016
Periscelididae	2	3	8	5	all Boreal and Maritime ecozones, Montane Cordillera, Prairies, Mixedwood Plains	Mathis and Rung 2011
Odiniidae	5	6	8	3	all Boreal and Maritime ecozones, Montane Cordillera, Prairies, Mixedwood Plains	Gaimari and Mathis 2011
Opomyzidae	10	11	11	4	all ecozones	Wheeler et al. 1999
Aulacigastridae	3	2	3	1	all but Arctic	Rung and Mathis 2011
**Superfamily Carnoidea**						
Chloropidae	100	140	361	260	all ecozones	
Milichiidae	11	13	55	20−30	all ecozones	Brake 2009, Brochu and Wheeler 2009
Canacidae	5^15^	10	11	0	all ecozones	Munari and Mathis 2010
Carnidae	8	12	21	5−10	all ecozones	Brake 2011, Stuke 2016
Acartophthalmidae	2	1	1	1	all but Arctic	
**Superfamily Ephydroidea**						
Drosophilidae	60	79	102	20−25	all ecozones	Brake and Bächli 2008, Miller et al. 2017, Bächli 2018
Ephydridae	150	197	182	10−15	all ecozones	Mathis and Zatwarnicki 1995
Curtonotidae	1	1	1	0	Prairies, Boreal Plains, Mixedwood Plains	Klymko and Marshall 2011
Diastatidae	5	7	11	2−3	all ecozones	Mathis and Barraclough 2011
Camillidae	1	2	1	0	Mixedwood Plains	
Braulidae	1	1	0	0	Prairies	
**Superfamily Sphaeroceroidea**						
Sphaeroceridae	35	184	190	20	all ecozones	Roháček et al. 2001
Heleomyzidae	74^16^	72	74	38	all ecozones	
Chyromyidae	5	5	10	5	Pacific Maritime, Prairies, Boreal Shield, Mixedwood Plains, Atlantic Maritime	
**Schizophora: Calyptratae**						
**Superfamily Hippoboscoidea**						
Hippoboscidae	13^17^	17	1	0	all but Arctic	Graciolli et al. 2007
‘**Muscoid grade**’						
Fanniidae	?^18^	84	85	8−10	all ecozones	
Muscidae	525^18^	440	479	40	all ecozones	
Anthomyiidae	375	515	412	10−30	all ecozones	Griffiths 1982–2004
Scathophagidae	130	126	115	29	all ecozones	
**Superfamily Oestroidea**						
Calliphoridae	40	62	39	0	all ecozones	
Oestridae	15	17	7	0	all ecozones	
Rhinophoridae	2	2	3	1	Boreal Shield, Newfoundland Boreal, Mixedwood Plains, Atlantic Maritimes	O’Hara et al. 2015
Sarcophagidae	85	135	132	5−15	all ecozones	
Tachinidae	500	736	647	100	all ecozones	O’Hara and Wood 2004
**Total**	**7056**	**9620**	**29,583**	**5205**−**20,458**		

^1^Higher classification follows a consensus of opinions of co-authors and collaborators; family limits follow Pape et al (2011). ^2^Numbers compiled from published records and collection holdings. ^3^Barcode Index Number as defined in Ratnasingham and Hebert (2013). ^3A^See figure 1 in Langor (2019) for a map of ecozones. ^4^McAlpine et al. (1979) included Cylindrotomidae, Limoniidae and Pediciidae in the Tipulidae.^5^McAlpine et al. (1979) included Corethrellidae in Chaoboridae. ^6^McAlpine et al. (1979) included this species in the Synneuridae which has since become a subfamily of the Canthyloscelidae. ^7^McAlpine et al. (1979) included Hesperinidae in Bibionidae. ^8^McAlpine et al. (1979) included Ditomyiidae, Bolitophilidae, Keroplatidae and Diadocidiidae in the Mycetophilidae. ^9^McAlpine et al. (1979) included Bolbomyiidae in Rhagionidae. ^10^McAlpine et al. (1979) included Mythicomyiidae in Bombyliidae. ^11^McAlpine et al. (1979) included *Iteaphila* group, Oreogetonidae, Brachystomatidae and Hybotidae in Empididae. ^12^McAlpine et al. (1979) included the Microphorinae and Parathalassiinae in the Empididae. ^13^McAlpine et al. (1979) included the Helcomyzidae and Heterocheilidae in the Dryomyzidae. ^14^McAlpine et al. (1979) included these species under Otitidae, now replaced by the senior valid name Ulidiidae. ^15^McAlpine et al. (1979) included 4 species in the Tethinidae which has since become a subfamily of Canacidae. ^16^McAlpine et al. (1979) included 4 species in the Trixoscelidinae which has since become a subfamily of Heleomyzidae. ^17^McAlpine et al. (1979) included 1 species in the Nycteribiidae and 1 in the Streblidae, both of which have since become subfamilies of Hippoboscidae. ^18^McAlpine et al. (1979) included Fanniidae in Muscidae.

## Nematocerous Diptera

The nematocerous Diptera (Lower Diptera), previously known as Nematocera, include those species of adult flies with elongate antennae composed of at least four flagellomeres. The group includes 36 extant families worldwide, of which 33 occur in Canada. The concepts and names of many families have changed since JF McAlpine et al. (1979) (see below and Table 1).

As adults, nematocerous Diptera tend to be long-legged and, compared to brachyceran Diptera, weaker fliers. Larvae are found in a wide array of habitats and include a large number of aquatic and semiaquatic taxa (see Tipulomorpha and Culicomorpha below), fungal feeders, gall makers, detritus feeders, predators, and even parasites, among others. The biting flies are mostly in the Culicomorpha and include those species that vector important diseases of humans, domestic animals and wildlife.

The nematocerous Diptera are clearly paraphyletic in relation to the Brachycera, although the exact sister group of Brachycera within the nematocerous Diptera is not certain (Woodley et al. 2009). The phylogenetic relationships among families have also been, in part, rather unstable. The phylogenetic analysis by Wood and Borkent (1989) laid groundwork, which was largely supported by Oosterbroek and Courtney (1995). Michelsen (1996) proposed the Neodiptera, a clade including Axymyiidae, Pachyneuridae, Bibionidae, Sciaroidea, Perissommatidae, Scatopsoidea, Anisopodidae, and Brachycera based on characters of the adult prothorax and cervical sclerites. However, a study of the male internal reproductive system by Sinclair et al. (2007) did not support the Neodiptera and indicated instead that the Blephariceridae + Psychodidae + Trichoceridae + Anisopodidae + Brachycera formed a monophyletic assemblage.

Molecular analyses have proposed a wide array of differing relationships that conflict with each other, at least in part, and with most morphological analyses (Pawlowski et al. 1996, Friedrich and Tautz 1997, Miller et al. 1997, Bertone et al. 2008). Wiegmann et al. (2011) and Lambkin et al. (2013) have provided the most recent overall interpretation of family relationships based on both morphological and molecular evidence, but these have major issues of interpretation (Borkent 2018). See below for summaries of the limits and phylogeny of the infraorders.

### Infraorder Tipulomorpha (F Brodo)

The major change to this infraorder since JF McAlpine et al. (1979) is the division of the Tipulidae into four families: Tipulidae, Cylindrotomidae, Limoniidae, and Pediciidae. Most European workers had recognized the family status of the first three taxa for decades, as Byers (1992) carefully documented while still favouring the inclusion of all craneflies in a single family. Starý (1992) elevated the pediciines from a tribe of the limoniines to full family status. The recognition of four families of crane flies remains a contentious issue among taxonomists. Molecular analyses (Bertone et al. 2008, Wiegmann et al. 2011) as well as a recent morphological study (Lukashevich and Ribeiro 2018) indicate that Limoniidae are paraphyletic, thereby calling into question the family ranking of these crane fly taxa. In the present work we have decided to follow the four-family concept, mostly to remain aligned with the classification used in the online Catalogue of the Craneflies of the World (Oosterbroek 2018) and BOLD. Tipulomorpha also include Trichoceridae (winter crane flies), a small family now formally recognized as the sister group to the Tipulidae s. lat. (crane flies) (Bertone et al. 2008, Wiegmann et al. 2011, Wiegmann and Yeates 2017).

Most recent taxonomic work in this infraorder has focused on the crane flies, bringing the total number of Canadian species to 633, mainly in the families Limoniidae (354 species in Canada) and Tipulidae (216), representing an increase of 21% since JF McAlpine et al. (1979) (Table 1). Monographs of *Chionea* Dalman (Byers 1983), *Dicranoptycha* Osten Sachen (Young 1987) and *Symplecta* (*Symplecta* Meigen) (Starý and Brodo 2009) in the Limoniidae and of *Nephrotoma* Meigen (Tangelder 1983, Oosterbroek 1984), *Prionocera* Loew (Brodo 1987), and *Tipula* (*Eremotipula* Alexander) (Gelhaus 2005) in the Tipulidae, have added species to our fauna, as did the documentation of crane flies of the Canadian Arctic (Brodo 1990, 2000) and additions to the eastern Canadian aquatic crane flies (LeSage and Harrison 1981, Sinclair 1988, Gathmann and Williams 2006). There are nearly as many BINs as there are recorded species of crane flies in Canada, although not every species has been barcoded, and it is expected that over 200 additional species will eventually be documented, mostly in the Pediciidae (Table 1). The number of Trichoceridae (21) has not changed much since 1979, but 10 more species are expected in Canada (Table 1).

Crane flies are mostly aquatic or semi-aquatic but a few, notably the pest species, are terrestrial and associated with roots of grasses and herbaceous plants. Many larvae are saprophagous, fungivorous, (*Limonia* Meigen and *Metalimnobia* Matsumura species), or carnivorous (some Limoniidae and Pediciidae species), and Cylindrotomidae are phytophagous. *Tipulapaludosa* Meigen and *T.oleracea* (Linnaeus) are established pests of dairy lands and golf courses (Gelhaus 2001). The larvae of winter crane flies feed on detritus and fungi and are often associated with small animal burrows or bird’s nests (Dahl 1973).

#### Nymphomyiidae and Deuterophlebiidae (BJ Sinclair)

The placement of these two families remains controversial. Previously, they have both been assigned to the infraorder Blephariceromorpha (Wood and Borkent 1989, Oosterbroek and Courtney 1995), Nymphomyiidae alone as sister to the Culicomorpha (Sæther 2000), and one or both families as sister to all Diptera (Bertone et al. 2008, Wiegmann et al. 2011, Lambkin et al. 2013, Sinclair et al. 2013). Deuterophlebiidae are often considered sister to Blephariceridae (Wood and Borkent 1989, Oosterbroek and Courtney 1995) or possibly sister to Nymphomyiidae (Schneeberg et al. 2011, 2012). With the generally accepted assignment of Blephariceridae to the re-defined Psychodomorpha (see below), we have chosen not to assign these two families to infraorder.

Only one species of minute Nymphomyiidae (<2 mm long) is recorded from Quebec and New Brunswick (Courtney 1994). The second nymphomyiid species listed in JF McAlpine et al. (1979) was re-interpreted and transferred to Chironomidae (Kevan and Cutten 1981). The Deuterophlebiidae were revised by Courtney (1990) and are confined to the mountains of western Canada where three species are known and another expected (Table 1). Both families have aquatic immature stages that are morphologically adapted to fast-flowing waters.

### Infraorder Psychodomorpha (G Curler and BJ Sinclair)

The limits of the Psychodomorpha have either been based on adult thoracic features (Hennig 1973, JF McAlpine et al. 1979) or defined by a suite of larval characters (Wood and Borkent 1989). The latter grouping has been viewed as a heterogeneous assemblage of non-Neodiptera (Psychodidae, Trichoceridae) and Neodiptera (Perissommatidae, Anisopodidae and Scatopsoidea) families (Michelsen 1996). Recent analyses support a three-family concept, namely Blephariceridae, Psychodidae and Tanyderidae (Bertone et al. 2008, Wiegmann et al. 2011); however, these families did not form a clade in the analyses in Lambkin et al. (2013). Additional support for a relationship between Psychodidae and Tanyderidae is based on wing venation (Bertone et al. 2008, Borkent and Sinclair 2012). The three family concept of this infraorder is followed here.

The number of species of Blephariceridae known from Canada (7) has decreased from the 1979 estimate, due to several synonymies (Hogue 1987, Courtney 2000), and three more species are expected (Table 1). Two of the four Nearctic species of Tanyderidae occur in Canada and one more may eventually be found here (Table 1). The immatures of blephacerids are conspicuously adapted to fast-flowing waters while those of the Tanyderidae occur in slower moving streams. The larval biology and morphology of western tanyderid species are documented in Wipfler et al. (2012).

The Psychodidae fauna of Canada is known to include three subfamilies, 15 genera and 34 species (Quate 1955, Young and Perkins 1984, G Curler unpubl. data; Table 1). Phlebotominae and Trichomyiinae are represented by three and one species, respectively; all other records are Psychodinae. There are more than three times as many BINs as recorded species in this family indicating that a relatively large number of species (we estimate 10–50) remain to be documented (Table 1). Species of *Lutzomyia* França are hematophagous and mainly tropical or subtropical, with Canadian records representing the northernmost limits in the western Hemisphere for Phlebotominae. Most species of Nearctic Psychodinae are detritivores living among moist decaying plant material or in madicolous habitats along stream margins, headwaters or seeps. In addition, several species of Psychodinae occur in homes and other habitats with anthropogenic influence (e.g., sewage treatment facilities, latrines, farmyards, polluted drainages).

### Infraorder Ptychopteromorpha (BJ Sinclair)

Wood and Borkent (1989) proposed the infraorder Ptychopteromorpha for two small families of flies, Ptychopteridae and Tanyderidae. Molecular and morphological evidence supporting the transfer of the Tanyderidae to the Psychodomorpha (Bertone et al. 2008, Borkent and Sinclair 2012), has resulted in this infraorder being represented solely by the family Ptychopteridae. The phylogenetic placement of this infraorder among the nematocerous Diptera remains disputed, although the multi-chambered male accessory glands are similar to those of Bibionomorpha and Culicomorpha (Sinclair et al. 2007). Currently four Canadian species in Bittacomorphinae and four species in Ptychopterinae are known for this entirely aquatic lineage, but an additional eight species are expected to eventually be found in the country (Table 1). The North American species of the subfamily Bittacomorphinae have recently been revised and keys to species provided (Fasbender and Courtney 2017).

### Infraorder Culicomorpha (A Borkent and DC Currie)

This infraorder includes eight families, all of which occur in Canada (Table 1). This is one more than recognized in JF McAlpine et al. (1979) due to the subsequent recognition of Corethrellidae as distinct from the Chaoboridae (Wood and Borkent 1989). Phylogenetic relationships among the families of Culicomorpha are well known and have considerable support (Borkent 2012, Kutty et al. 2018) but the position of Chironomidae needs further testing, as either the sister group of all remaining families, or as the sister group of Ceratopogonidae.

With 798 named species, the Chironomidae (non-biting midges) currently stand as the most species-rich family of Diptera in Canada, and at least 1000 additional species are expected to occur in the country (Table 1). The remaining families of non-biting midges, namely Chaoboridae (11 species in Canada), Thaumaleidae (13) and Dixidae (34) are represented by relatively few species (Table 1). Focused collecting at microhabitats of the latter two families has greatly increased the number of Canadian records (Moulton 2017, Pivar et al. 2018) and as many as 15 additional species are expected to occur in the country.

The remaining four families have some or all species with biting females. The Culicidae (82 species in Canada), Simuliidae (164) and Ceratopogonidae (263) are all quite diverse, whereas a single species of Corethrellidae (formerly in Chaoboridae) is known for the country (Table 1). The Culicidae (mosquitoes) and Simuliidae (black flies) are both very well known, but while only three additional mosquito species are estimated to be unrecorded, a further 20 species of black flies are expected to eventually be documented in Canada (Table 1). The Ceratopogonidae (biting midges) are by far the most poorly known of biting flies. There are close to ten times as many BINs as recorded species for the group and it is thought that less than half of the Canadian fauna is known to date (Table 1).

The medical and veterinary significance and dominant presence in aquatic systems of so many Culicomorpha has meant that they are some of the best known of the Diptera, including interpretation of their immatures. Taxa may be identified using the following references: Chaoboridae (Sæther 1972, larvae, pupae, adults), Corethrellidae (Borkent 2008, adults; McKeever and French 1991, larvae, pupae), Culicidae (Mattingly 1971, larvae, pupae, adults to genus; Wood et al. 1979, larvae, adults) Chironomidae (Wiederholm 1986, 1989, pupae, adults to genus; Andersen et al. 2013, larvae to genus), Ceratopogonidae (Downes and Wirth 1981, adults to genus; Borkent 2014, pupae to genus), Simuliidae (Adler et al. 2004, larvae, pupae, adults), and Thaumaleidae (Arnaud and Boussy 1994, Pivar et al. 2018, Sinclair 1996, adults). Dixidae are poorly understood and require fundamental revision (Greenwalt and Moulton 2016).

Immature Culicomorpha are aquatic in both lotic and lentic habitats where they are prey for aquatic organisms, including fish. The Chironomidae are especially common, occupying virtually every aquatic niche, including tree holes, rivers, lakes, and even tidal habitats where their abundant larvae often have a strong influence on aquatic community structure. As adults, the Culicidae are the most prevalent, ubiquitous and persistent blood feeders in Canada, where some species are vectors of arboviruses, including West Nile virus, currently the most common mosquito-borne infection of humans in the country (Roth et al. 2010). Simuliidae are also quite common and sometimes very abundant in large rivers and lake outlets, with the resulting blood feeding activities affecting both humans and livestock. Most Ceratopogonidae are predaceous but the majority of *Culicoides* Latreille species have biting females. One species, *C.sonorensis* Wirth and Jones, is a vector of Bluetongue virus of cattle and other ruminants in south-central British Columbia (Sellers and Maarouf 1991). Female Corethrellidae are known only to bite frogs.

#### Axymyiidae (BJ Sinclair)

The phylogenetic relationships and systematic assignment of the family remains disputed (Sinclair 2013). It has been variously assigned to a monotypic Axymyiomorpha due to the absence of synapomorphies (Wood and Borkent 1989, Borkent and Sinclair 2012, Ševčík et al. 2016), to a variably defined Bibionomorpha (Oosterbroek and Courtney 1995, Grimaldi and Engel 2005, Pape et al. 2011), to Axymyiomorpha (incl. Axymyiidae, Perissommatidae, *Pachyneura* Zetterstedt) (Amorim 1993), or considered a sister family to Bibionomorpha s. str. (Wiegmann et al. 2011, Sinclair et al. 2013).

Axymyiidae are a small family of Holarctic flies with a single eastern species, *Axymyiafurcata* McAtee, recorded from Canada (Ontario, Quebec) but two species from the Pacific Northwest (Sinclair 2013, Fitzgerald and Wood 2014) are expected to occur in British Columbia (Table 1). Detailed descriptions of all life stages and keys to Nearctic species are provided by Wood (1981), Wihlm et al. (2012), and Fitzgerald and Wood (2014). The life history of the eastern Nearctic *A.furcata* is well documented (Wihlm and Courtney 2011) and all known larval stages in Axymyiidae are restricted to burrowing in water-permeated wood.

### Infraorder Bibionomorpha s. lat. (BJ Sinclair)

The boundaries of the Bibionomorpha have revolved around the nematocerous families included in the Neodiptera by Michelsen (1996), but there has been little consensus. Hennig (1973) favoured a broad concept that included the Bibionidae, Pachyneuridae, Sciaroidea, Scatopsoidea, Anisopodidae, Axymyiidae, and Perissommatidae (non-Nearctic), whereas Wood and Borkent (1989) restricted the infraorder to Bibionidae, Pachyneuridae, and Sciaroidea. Amorim (1993) included the following groups in the Bibionomorpha: Bibionidae, Pachyneuridae (in part), Sciaroidea, and Anisopodidae. More recently molecular analyses have again supported the broad concept sensu Hennig (1973), exclusive of Perissommatidae (Wiegmann et al. 2011) or exclusive of both Perissommatidae and Axymyiidae (Bertone et al. 2008). Grimaldi and Engel (2005) also recognized a broad concept, although exclusive of Scatopsoidea. Recently Sinclair et al. (2013) showed that the male terminalia of Perissommatidae show derived attributes of the Bibionomorpha s. str. Given these conflicting classifications we have chosen to use both narrow (s. str.) and broad (s. lat.) concepts of the group as followed in Ševčík et al. (2016). The phylogenetic relationships within the Bibionomorpha s. str. have been studied by Wood and Borkent (1989), Amorim and Rindal (2007), and Ševčík et al. (2016).

Three families are excluded from Bibionomorpha s. str. due to the absence of a highly modified and multi-chambered accessory gland and different configuration of the ejaculatory apodeme (Sinclair et al. 2013). Anisopodidae and Canthyloscelidae (= Synneuridae) have low diversity in Canada (six and one species, respectively) but the Scatopsidae are represented by 30 species, with 15–20 more expected (Table 1). Canadian Scatopsidae can be identified using the genus key in Cook (1981) and species keys referred to therein. The Anisopodidae have not received much recent taxonomic attention in the Nearctic, although species of *Sylvicola* Harris were revised by Pratt and Pratt (1980). The larvae of these families are saprophagous and found in moist decaying organic matter.

### Infraorder Bibionomorpha s. str. (BJ Sinclair)

Seven of the ten families of Bibionomorpha s. str. found in Canada are species-poor, including Pachyneuridae (1 species in Canada), Bibionidae (26), Hesperinidae (1; formerly in Bibionidae), and the following four families, formerly included in Mycetophilidae by J.F. McAlpine et al. (1979): Ditomyiidae (3), Bolitophilidae (16), Keroplatidae (28) and Diadocidiidae (2) (Table 1). The number of species of Bibionidae known from Canada has decreased by approximately a third from the 1979 estimate (Table 1), primarily due to numerous synonyms discovered subsequently (S Fitzgerald pers. comm.). For all families, a few additional species are eventually expected to be found in Canada.

The remaining three families are much more diverse. The number of Sciaridae (129 species in Canada) has quadrupled since 1979 (Table 1), primarily due to ongoing revisionary studies by Scandinavian and German colleagues (see Mohrig et al. 2013 and subsequent papers by these authors). The numbers of known Cecidomyiidae (243 species in Canada) have more than doubled since 1979 and those of Mycetophilidae now reach 489 species, thereby representing the highest documented diversity of any family in this infraorder (Table 1). Diversity estimates based on BINs are all much higher than the known fauna for these three families, and especially for the Cecidomyiidae, where they suggest that the known species represent only approximately 2% of the Canadian fauna (Table 1). Hebert et al. (2016) estimated 16,000 species of Cecidomyiidae in Canada, a 10-fold increase from the diversity predicted in 1979, a remarkable figure, but one consistent with the newly appreciated diversity of this family in temperate (Jaschhof and Jaschhof 2009, 2013) and tropical (Borkent et al. 2018, Brown et al. 2018) sites. As we are uncertain whether BINs indicate number of species, it is difficult to predict the number of species in Canada and it is possible that anywhere from 1000–16,000 species remain to be documented in the country. The great diversity of this family is in part due to the apparent host specificity of plant-feeding species, with several of economic importance. Hundreds of species of sciarids and mycetophilids also await documentation in Canada (Table 1).

Although knowledge of the species diversity of Cecidomyiidae appears sparse, general information and identification to genera of the subfamily Cecidomyiinae are provided by Gagné (1989, 2018). The genera of Mycetophilidae can be keyed in Vockeroth (1981), but some subfamilies are now recognized as families (see above). Several genera of Mycetophilidae have been revised since 1979, including: *Acomoptera* Vockeroth (Kerr 2011), *Leptomorphus* Curtis (Borkent and Wheeler 2012), *Mycomya* Rondani (Väisänen 1984), *Phthinia* Winnertz (Fitzgerald and Kerr 2014), *Sciophila* Meigen (Zaitzev 1982) and *Trichonta* Winnertz (Gagné 1981).

Members of the Bibionomorpha s. str. are most abundant in moist woodlands, with many larvae found in fungi, in dead wood and other decaying plant material, beneath bark, and in a variety of other microhabitats. The majority of Cecidomyiidae are associated with plants, forming galls or developing in flowers and leaf rolls, whereas others are inquilines on plant hosts damaged by other gall midges. Some are also associated with fungi, or free-living predators. A number of species of Cecidomyiidae are serious pests of cereals, Brassicaceae, conifers, apple trees, etc., and a zoophagous species is used in the biocontrol of aphids (Darvas et al. 2000).

## Suborder Brachycera: Lower Brachycera

Brachycera are a monophyletic suborder traditionally defined by a short antenna with a modified flagellum (third antennal segment) made up of 3–8 fused flagellomeres. The group is very diverse with 83 families occurring in Canada (Table 1). The Brachycera are usually divided into the paraphyletic Lower Brachycera and the monophyletic Eremoneura, which contains both the monophyletic Empidoidea and Cyclorrhapha.

The Lower Brachycera are a large and undoubtedly unnatural assemblage of mostly large and conspicuous flies. Until recently, this group was widely referred to as the Orthorrhapha, but morphological and molecular evidence indicate that it is paraphyletic, at least with respect to the Cyclorrhapha (Woodley 1989). With the assignment of the Empidoidea to the Eremoneura (which includes both Cyclorrhapha and Empidoidea) (Griffiths 1972), the term Lower Brachycera is now used to refer to the non-Eremoneuran Brachycera. Considerable research globally has focused on this group, especially among the therevoid clade (e.g., Woodley 1989, Sinclair et al. 1994, Yeates 1994, 2002, Wiegmann et al. 2000, 2011, Winterton et al. 2007, Trautwein et al. 2010, Shin et al. 2017). Division of the Lower Brachycera is largely stable, with well supported Xylophagomorpha (Woodley 1989), Tabanomorpha (Sinclair et al. 1994, Wiegmann et al. 2000, 2011, Kerr 2010), Stratiomyomorpha (Sinclair et al. 1994, Wiegmann et al. 2011) and Asilomorpha (Woodley 1989, Wiegmann et al. 2011). Several families remain difficult to assign phylogenetically and continue to float between infraorders, i.e., Acroceridae, Hilarimorphidae, Nemestrinidae. Major changes since JF McAlpine et al. (1979) include the recognition of a new family Oreoleptidae (Zloty et al. 2005) and elevation of the Bolbomyiidae (Kerr 2010) and Mythicomyiidae (Evenhuis 2002) from Rhagionidae and Bombyliidae, respectively.

### Infraorder Xylophagomorpha (BJ Sinclair)

This infraorder is represented by the single family Xylophagidae, although some authors have divided it into smaller family units (Woodley 1989). The Xylophagomorpha are considered the sister group to the Tabanomorpha in most analyses (Wiegmann et al. 2000, 2011, Sinclair et al. 2013, Shin et al. 2017). Five genera and 14 species are recorded from Canada (Woodley 2011c), similar to numbers recorded in 1979, and another one or two species are expected (Table 1). Current knowledge of the group, generic diagnoses, a key to world genera and catalogue of species have been compiled by Woodley (2011c). Xylophagids are found primarily in wooded and forest regions where the larvae are predators of wood inhabiting insects.

### Infraorder Tabanomorpha (BJ Sinclair)

Woodley (1989) and Sinclair et al. (1994) summarized the morphological evidence for relationships of the Tabanomorpha. Much of the uncertainty of higher level phylogeny of the Tabanomorpha is due to doubts concerning the limits and monophyly of the Rhagionidae. Through combined morphological and molecular analyses, Kerr (2010) redefined the family Rhagionidae, establishing its monophyly and recognizing the families Austroleptidae (Australia and Chile) and Bolbomyiidae. The classification of Kerr (2010) is followed here.

Six families of Tabanomorpha occur in Canada and these are organized in two superfamilies. In Rhagionoidea, Bolbomyiidae include three known species in Canada and two more are expected (Table 1). Rhagionidae include 48 species, a substantial increase from 1979, and 10–15 additional species are expected (Table 1). No modern species keys are available for the large genera in the Rhagionidae and most of the family is in need of revision. Adults are common in forested regions, where most larvae occur in damp forest litter and beneath mats of mosses. The immature stages of the Bolbomyiidae are unknown.

In Tabanoidea, three families have low diversity: Pelecorhynchidae (5 species in Canada), Athericidae (2), and the recently erected monotypic family Oreoleptidae (1) (Zloty et al. 2005; Table 1). In part due to their large size and the biting habits of most females, the Tabanidae (horse flies and deer flies) are much better known than most insects in Canada, with keys and illustrations known for all 142 species (Teskey 1990, Thomas and Marshall 2009, Thomas 2011) as well as a complete catalogue (Burger 1995). With the slight increase in species richness since 1979, this family is now considered to be very well known and no additional species are expected in Canada. The larvae of Athericidae and Oreoleptidae occur in riffle zones and/or vegetation of cool streams and flowing rivers. Those of Pelecorhynchidae and Tabanidae are predators of invertebrates found mostly in wetland soils.

### Infraorder Stratiomyomorpha (M Hauser)

The infraorder Stratiomyomorpha includes three families, of which the Stratiomyidae and the Xylomyidae occur in Canada while the Pantophthalmidae are restricted to the Neotropics. The sister-group relationship of Stratiomyidae and Xylomyidae is strongly supported, especially by larval characters (Woodley 1989).

The Stratiomyidae are represented by 114 species in Canada, a substantial increase from 1979 (Table 1), and 5–10 more are expected (Table 1). At least five species of Stratiomyidae have been introduced from Europe, Australia and the USA (Swann et al. 2006, Marshall et al. 2015). The fauna is rather well known, although revisions are needed especially for groups with aquatic larvae (*Caloparyphus* James, *Stratiomys* Geoffroy, *Odontomyia* Meigen, *Nemotelus* Geoffroy), which could reveal a few undescribed species as well as some synonyms. Only two genera and seven species of Xylomyidae are known from Canada (Woodley 2011b) but numbers have nearly doubled since 1979 (Table 1); a key to the Canadian species is provided by Webb (1984).

Stratiomyids are usually found in humid and forested areas where their larvae are terrestrial or aquatic, feeding mostly on decaying plant and animal materials (Woodley 2001). The larvae of Xylomyidae are found under the bark of trees but little is known of the biology of these uncommon flies.

#### Acroceridae and Nemestrinidae (BJ Sinclair)

Both families have been assigned to the Nemestrinoidea based on the parasitic larvae with hypermetamorphosis (Woodley 1989), but this infraorder (including Bombyliidae) is now generally considered polyphyletic with the three parasitic families considered to be distantly related (Yeates 1994, 2002, Winterton et al. 2007, Wiegmann et al. 2011, but see Shin et al. (2017) for a divergent opinion). Only two species of Nemestrinidae are found in Canada (Table 1), the same as in 1979, and these are confined to the central arid regions of British Columbia. Twenty species of Acroceridae are recorded from Canada; a key to New World genera is available in Schlinger et al. (2013), but only one recent revision has included Canadian records (Borkent et al. 2016). A few more acrocerid species are therefore expected in the country. The larvae of Acroceridae are internal parasites of spiders, whereas those of Nemestrinidae are parasitic on grasshoppers and beetles.

### Infraorder Asilomorpha (BJ Sinclair)

The higher classification and phylogeny of the Asilomorpha (containing one superfamily – Asiloidea) has received a great deal of focus over the past decades (e.g., Woodley 1989, Winterton et al. 2007, Trautwein et al. 2010, Winterton and Ware 2015). Discussion of the limits of the Asilomorpha, which appear paraphyletic in relation to Eremoneura (Sinclair et al. 1994, Trautwein et al. 2010), has primarily revolved around the placement of the genus *Hilarimorpha* Schiner, which has previously been assigned to the Bombyliidae, Therevidae, or its own separate family considered sister to the Bombyliidae, Asiloidea or Eremoneura (Trautwein et al. 2010).

Eight families of generally large and showy Asilomorpha occur in Canada, five of which are relatively species-poor, including Apioceridae (1 species in Canada), Mydidae (2), Mythicomyiidae (1), Hilarimorphidae (7), and Scenopinidae (10) (Table 1). The diversity of Apioceridae and Mydidae in the southern interior of British Columbia was documented by Cannings (2006), whereas the Mythicomyiidae, Hilarimorphidae and Scenopinidae are poorly documented and more species are expected in Canada (Table 1).

The Canadian fauna of the larger families of Asilomorpha has received much attention since JF McAlpine et al. (1979). The number of Asilidae (222 species in Canada) has nearly doubled, primarily through the recent publications of Cannings (1994, 1997, 2002), and a few additional species may eventually be added (Table 1). The diversity of the Bombyliidae in Canada is outlined in a world catalogue (Evenhuis and Greathead 1999), but the Canadian fauna had largely been ignored for decades until the publication of an illustrated key to eastern Canadian species (Kits et al. 2008). The 105 species of Bombyliidae currently documented represent a 50% increase since 1979 and more than 40 additional species are expected in Canada (Table 1). A world catalogue of Therevidae outlines the Canadian diversity of this family (Webb et al. 2013). With 50 species now recorded from Canada, this family is very well known and no additional species are expected (Table 1).

The Asilomorpha display a wide range of habitats and life histories. The Scenopinidae have predaceous larvae associated with wood-boring larvae, bird’s nests, and carpet beetle larvae. The larvae of Hilarimorphidae are unknown and adults are sporadically collected, with verified records indicating that they frequent riverbanks. Adult Mythicomyiidae are flower visitors, feeding on pollen and nectar, whereas the few larval observations suggest egg pod predators of grasshoppers and inquilines in ant nests. Adult Asilidae are efficient predators with highly modified mouthparts; the larvae live in soils and rotting wood. Larvae of Therevidae are often found burrowing through sandy soils (Irwin and Lyneborg 1981). The larvae of Mydidae and Apioceridae are predaceous in sandy soils and adults are flower feeders. The Bombyliidae are generally parasitic on various Holometabola or predaceous on egg pods of grasshoppers (Hall 1981), with adults visiting flowers.

## Suborder Brachycera: Eremoneura

The monophyly of Eremoneura is strongly supported and the group comprises the monophyletic Empidoidea and Cyclorrhapha (Cumming et al. 1995, Sinclair and Cumming 2006, Wiegmann et al. 2011, Lambkin et al. 2013). The Eremoneura now also include the monotypic Nearctic family Apystomyiidae (not in Canada), which is considered to be either the sister group of Cyclorrhapha (Trautwein et al. 2010, Wiegmann et al. 2011), or the sister group of the entire Eremoneura (Sinclair et al. 2013). The Eremoneura as a group was not recognized in JF McAlpine et al. (1979).

### Superfamily Empidoidea (JM Cumming and BJ Sinclair)

The Empidoidea are a monophyletic lineage comprising five main families, namely Atelestidae, Brachystomatidae, Empididae, Hybotidae, Dolichopodidae s. lat. (including Microphorinae and Parathalassiinae) (Sinclair and Cumming 2006) and three previously unassigned genus-groups. Some authors have treated two of these genus-groups as separate families (i.e., Homalocnemidae (non-Nearctic) and Oreogetonidae), because of the availability of family-group names (Thompson 2009, Pape et al. 2011, Marshall 2012). The *Iteaphila* group has recently been elevated to subfamily rank within yet another newly recognized Empidoidea family, Ragadidae (Wahlberg and Johanson 2018). Recognition of this family is controversial and generally not accepted by the empidoid community, nor is it accepted herein.

Five families of this primarily predaceous group occur in Canada (Table 1): Oreogetonidae (7 species), Empididae (251), Brachystomatidae (11), Hybotidae (155), and Dolichopodidae s. lat. (508), plus the *Iteaphila* genus-group (17). Apart from the Dolichopodidae (exclusive of Microphorinae and Parathalassiinae), the remaining groups were lumped into the Empididae in JF McAlpine et al. (1979). The current total of 949 Canadian species of Empidoidea is a moderate increase over the 800 species recorded by JF McAlpine et al. (1979). Many empidoid genera still require study and recent Nearctic revisions (e.g., Sinclair et al. 2011, Sinclair and MacDonald 2012, Brooks and Cumming 2017) have resulted in numerous new species descriptions. The key to the Nearctic genera of Empididae in Steyskal and Knutson (1981) follows the family concept used by JF McAlpine et al. (1979) and is now out-of-date. Hundreds of additional empidoid species are expected to be eventually documented in Canada (Table 1).

The Oreogetonidae and two subfamilies of Empididae (Clinocerinae and Hemerodromiinae) include species with aquatic larvae. The remaining Empididae are mainly terrestrial and many species are important pollinators (Rader et al. 2016), especially in alpine and arctic regions (Lefebvre et al. 2014). The Hybotidae are common predators in forests, grasslands and agricultural fields (Sinclair and Cumming 2017), whereas the Dolichopodidae are significant predators in various aquatic, semi-aquatic and terrestrial habitats (Grichanov and Brooks 2017).

## Suborder Brachycera: Eremoneura: Cyclorrhapha

The Cyclorrhapha constitute the most diverse lineage of Brachycera and include the numerous families of higher flies that pupate inside the last larval exuviae (i.e., puparium). The group is divided into the basal Lower Cyclorrhapha (“Aschiza”) and the monophyletic Schizophora (i.e., flies with a protrusible ptilinum for exiting the puparium). Schizophora are further divided into the paraphyletic Acalyptratae and the monophyletic Calyptratae.

### Lower Cyclorrhapha (“Aschiza”)

No recent hypotheses support the monophyly of the Aschiza, which traditionally included the cyclorrhaphan families exclusive of Schizophora (or those flies without a ptilinum for exiting the puparium). Only Brown (1992, 1995) and Disney (1994) have supported the monophyletic Aschiza concept proposed by McAlpine (1989). All other morphological and molecular analyses have shown that the “Aschiza” are a grade and should be referred to as the Lower Cyclorrhapha (Griffiths 1972, Cumming et al. 1995, Zatwarnicki 1996, Collins and Wiegmann 2002, Moulton and Wiegmann 2004, Wiegmann et al. 2011, Pauli et al. 2018). This is an important lineage to understand phylogenetically as it sets the stage for the massive radiation of Schizophora. Unfortunately, there has been a profound lack of agreement about relationships within this grade.

#### Lonchopteridae (J Skevington and JM Cumming)

Placement of Lonchopteridae has been one of the most intractable problems within Diptera phylogenetics. The family has floated around in different analyses, in some cases being proposed as sister to the rest of Cyclorrhapha (Griffiths 1972), as sister to the Phoroidea (Brown 1992, Cumming et al. 1995), or as sister to (or within) the Platypezoidea + Phoroidea (Collins and Wiegmann 2002, Moulton and Wiegmann 2003, Wiegmann et al. 2011). Seven species of lonchopterids are known from Canada (Klymko and Marshall 2008) and another could eventually be discovered. Larvae are found in wet, decaying organic matter where they feed on bacteria and fungi. Two species occur in aquatic environments such as springs, seeps and shorelines (Valliant 2002). Adults feed on fungi, nectar, pollen and dead insects (Klymko and Marshall 2008).

#### Superfamily Platypezoidea (J Skevington and JM Cumming)

The status of this superfamily is contentious and its use should probably be abandoned. In the strict sense it appears to include Platypezidae (including *Microsania* Zetterstedt and *Melanderomyia* Kessel) and Opetiidae (non-Nearctic) (Tkoč et al. 2017), but even this is controversial, as Opetiidae has also been placed outside the superfamily in numerous positions in various phylogenies. There are 39 named platypezid species known from Canada and while a few more species are still expected (Table 1), some recent synonymies were established (e.g., Cumming and Wheeler 2016) and more are likely to occur as previous revisionary work, primarily by EL Kessel and associates (e.g., Kessel and Buegler 1972), routinely described males and females as separate species. Immature platypezids are fungivorous and the males of many species form large swarms.

#### Superfamily Phoroidea (J Skevington and JM Cumming)

Phylogenetic analyses that include the relevant taxa support the relationship of Phoridae (including Sciadocerinae sensu Brown et al 2015, Disney 2001) and Ironomyiidae (non-Nearctic) in this superfamily (Wiegmann et al. 2011, Young 2018). There are 135 named species of Phoridae known from Canada with the diversity estimated to be much greater, with perhaps 300 additional species (B Brown pers. comm.; Table 1). Phorids likely have the widest diversity of larval lifestyles of any insect family. Although some of the most common species are decomposers (including carrion feeders), others are fungivorous, phytophagous (including leaf miners), inquilines in social insect nests, predators, or parasitoids.

#### Superfamily Syrphoidea (J Skevington)

This is another higher grouping that should likely be abandoned. Pipunculidae and Syrphidae have been proposed as sister taxa in all published morphological phylogenetic hypotheses (Sinclair et al. 2013 and references therein), but most molecular analyses refute this relationship and place Pipunculidae as sister to Schizophora and Syrphidae as sister to Pipunculidae + Schizophora (Wiegmann et al. 2011, Pauli et al. 2018). Only Moulton and Wiegmann (2004) have proposed that Syrphidae are sister to Schizophora and that Pipunculidae are sister to Syrphidae + Schizophora. Recently discovered morphological evidence based on metapleural characters (Tachi 2014) supports the sister-group relationship of Pipunculidae and Schizophora.

There are currently 539 described species of Syrphidae recorded in Canada, a modest increase since 1979, and another 34 species are thought to occur (Table 1). The number of Pipunculidae species in Canada has almost doubled since 1979 and currently totals 85 species, but much of the fauna remains to be discovered and at least another 170 species are expected to occur. Most adult syrphids are pollinators, but larvae range from predators of aphids and other soft-bodied insects, predators and parasitoids of ants, to saprophages in rotting wood, slime fluxes, and sewage. Most pipunculids are parasitoids of Auchenorrhyncha (Skevington and Marshall 1997) and *Nephrocerus* Zetterstedt are parasitoids of Tipulidae (Koenig and Young 2007).

### Schizophora: Acalyptratae

The Schizophora are a large monophyletic subgroup of Cyclorrhapha characterized by an inflatable sac-like ptilinum that temporarily extrudes from the head of the adult fly to allow emergence from the puparium. This exceedingly successful lineage contains 54 families in Canada, which are traditionally divided into the paraphyletic Acalyptratae and the monophyletic Calyptratae.

Acalyptratae are a large and heterogeneous assemblage of families circumscribed by the absence of characters used to define the Calyptratae. Many families are readily characterized by appearance or habit, but support for relationships amongst them has been elusive, likely because several lineages originated in a short period as part of an explosive radiation following the K-T extinction event 65mya (Wiegmann et al. 2011). As such, support for family-level relationships is often weak, with the exception of a few groups within Ephydroidea, Nerioidea, and Tephritoidea. Evolutionary reconstruction and superfamily composition has therefore been contentious and varied historically (JF McAlpine 1989, Yeates et al. 2007). Nine superfamilies are currently recognized, all of which occur in Canada, and the number of families in the country totals 44 (Table 1). The Canadian acalyptrate fauna is relatively well known, although it is likely that many species remain undescribed, especially amongst taxa with diminutive species.

#### Superfamily Diopsoidea (O Lonsdale)

Diopsoidea, historically called Nothyboidea by some, are a weakly supported cluster of families of low-to-medium species richness. Current superfamily definitions largely stem from a classification developed by Hennig (1958), refined in subsequent studies (Hennig 1965, 1973), and later elaborated upon by JF McAlpine (1989) and DK McAlpine (1997a, b), who suggested alternate superfamily placement for some families. The classification of JF McAlpine (1989) is followed here, although phylogenetic studies testing this system are ongoing and classification is expected to change. Strongylophthalmyiidae were once considered a subfamily of Tanypezidae by some, but Lonsdale (2013) found that these sister-taxa are best represented by a two-family system.

The superfamily includes nine families, four of which occur in Canada (Table 1): Psilidae (27 species), Diopsidae (2), Strongylophthalmyiidae (2), and Tanypezidae (1). The Canadian fauna of these families appears to be relatively well-known and only a few species have been added in recent decades, but work on Psilidae is still required. Revisions and keys to Nearctic genera and species are available for Diopsidae (Feijen 1989), Strongylophthalmyiidae (Barber 2006) and Tanypezidae (Lonsdale 2013, Knab and Shannon 1916). Nearctic Psilidae were treated in Melander (1920) and subsequent revisions treating species of Nearctic *Loxocera* Meigen are available in Capelle (1953) and Buck and Marshall (2006b). Buck and Marshall (2006a) partially revised *Psila* Meigen. Little is known of the life histories of non-pest species. Most taxa for which information is known appear to be saprophagous in damaged or decaying plant material, but some are primary invaders of plants, and a few of these are occasional crop pests.

#### Superfamily Nerioidea (O Lonsdale)

The Micropezidae are the only nerioid family known in Canada. Some authors, including Hennig (1958), treated a number of micropezid subfamilies as full families under the assumption that the stilt-legged flies (Neriidae) rendered them paraphyletic, but most contemporary authors now follow DK McAlpine (1975, 1998) in recognizing a broad monophyletic Micropezidae.

Only sixteen of the approximately 700 described species of Micropezidae occur in Canada, one of which is a recently introduced European *Micropeza* Meigen (Hoebeke and Wheeler 1994). Species numbers have otherwise remained unchanged since 1979 and no additional species are anticipated (Table 1). Adult Micropezidae generally display distinctive stilt-like mid and hind legs, and most of the relatively few species for which oviposition or larval habitats are known occur in rotting wood (Marshall 2012) and a variety of other decomposing materials.

#### Superfamily Sciomyzoidea (JF Gibson)

The families presently composing Sciomyzoidea were treated separately as Conopoidea and Sciomyzoidea by JF McAlpine et al. (1979) and JF McAlpine (1989). The inclusion of the orphaned family Conopidae within the Sciomyzoidea was supported by Wiegmann et al. (2011) and has been generally accepted since (e.g., Marshall 2012). Other taxonomic changes within the superfamily since 1979 include the recognition of the Helcomyzidae (1 species in Canada) and Heterocheilidae (1) as families distinct from Dryomyzidae (Malloch 1933, DK McAlpine 1991a, b), bringing the total number of families found in Canada to seven (Table 1). Members of the Sciomyzoidea are some of the largest acalyptrates, but species diversity is relatively low with fewer than 200 species in Canada. Current numbers of species have not changed much since JF McAlpine et al. (1979) for Coelopidae (4 species), Dryomyzidae (8), and Sepsidae (19). For Sciomyzidae (120) and Conopidae (42), species numbers in Canada have increased by approximately 40% since 1979 (Table 1). A few additional species are expected to eventually be documented in four of the seven families.

Knutson et al. (1986) produced a catalogue of North American Sciomyzidae. Most other recent faunistic and taxonomic work on sciomyzoid families has been global in nature but with relevance to the Canadian fauna: Coelopidae (Mathis and McAlpine 2011); Conopidae (Gibson and Skevington 2013); Dryomyzidae (Mathis and Sueyoshi 2011); Helcomyzidae (Mathis 2011a); Heterocheilidae (Mathis 2011b); Sciomyzidae (Knutson and Vala 2011). As well, western Canadian Conopidae were treated by Gibson (2017). There has been no work on Canadian Sepsidae. Complete life history and range are not known for most species, but some taxa (Conopidae, Sciomyzidae) are parasitoids. Restriction to marine coasts is also common within the group (Coelopidae, Helcomyzidae, Heterocheilidae, Dryomyzidae – *Oedoparena*).

#### Superfamily Lauxanioidea (JF Gibson)

The recent molecular analysis of Wiegmann et al. (2011) supported a monophyletic Lauxanioidea, including two families found in Canada, Lauxaniidae and Chamaemyiidae. The current number of reported species in Canada for Lauxaniidae (78) and Chamaemyiidae (35) represent modest increases since 1979, but a few more species of each family are likely to be recorded in the future (Table 1). Most of the Canadian Lauxanioidea fauna has not been revised since JF McAlpine et al. (1979). A notable exception is the revision of *Pseudodinia* Coquillet (Chamaemyiidae) by Barber (1985), which added ten species to the Canadian list and detailed much of the life history known for the group. Species of Lauxaniidae are suspected to be saprophagous as immatures while those of Chamaemyiidae are parasitoids of aphids and other Sternorrhyncha.

#### Superfamily Tephritidoidea (O Lonsdale)

The superfamily was divided by Korneyev (2000a) into a monophyletic “higher Tephritidoidea” and a paraphyletic “lower Tephritidoidea”, all eight families of which are represented in Canada (Table 1). Korneyev (2000b) provided analysis and discussion of the family-level and genus groupings within Tephritidae, but admitted that much remains to be investigated. The “higher” families are Ulidiidae (35 species in Canada), Platystomatidae (10), Pyrgotidae (3), and Tephritidae (122). The “lower” families are Piophilidae (31), Pallopteridae (9), Lonchaeidae (99) and Richardiidae (1). Recently, a phylogeny by Han and Ro (2016), based on molecular data, questioned this system, supporting Tephritidae as paraphyletic, finding Richardiidae to belong to the “higher” group of families and a sister-group relationship to the remaining families. The numbers of known species have been relatively constant since 1979, with the exception of the family Tephritidae, which has tripled (Table 1). A few more species are expected to be discovered in Canada for most families, especially for Lonchaeidae, Ulidiidae and Tephritidae.

Revisions of the Nearctic fauna are available for Pallopteridae (Malloch and McAtee 1924), Pyrgotidae (Steyskal 1978) and Tephritidae (Foote et al. 1994). The global Piophilidae were revised by JF McAlpine (1977), and Rochefort and Wheeler (2015) reviewed the Piophilidae of northern Canada. The *Manual of Nearctic Diptera*, including references therein, is the best recent resources for identification of Lonchaeidae (JF McAlpine 1987), Platystomatidae (Steyskal 1987b) and Ulidiidae (Steyskal 1987a). Most Canadian Platystomatidae belong to *Rivellia* Robineau-Desvoidy and are keyed in Namba (1956), and most eastern Canadian Tephritidae species are easily identified using Jackson et al. 2011.

Species of most families are very conspicuously patterned, especially on the wings, and are behaviourally fascinating with elaborate courtship rituals. Lonchaeidae and Piophilidae are darker and less “charismatic”, and much remains to be discovered of their biology. Many taxa are saprophagous as larvae, but less commonly predaceous in damaged or decaying plant vegetation, e.g., Pallopteridae (Teskey (1976); a few are primary invaders of plants and may be pestiferous, especially Tephritidae (DK McAlpine 1973, Norrbom and Korytkowski 2010, Marshall 2012). Piophilidae prefer animal matter in advanced states of decay (JF McAlpine 1977). A minority of Tephritidae are saprophages, parasitoids, inquilines or predators, and Pyrgotidae are parasitoids of scarab larvae (Korneyev 2000b, Marshall 2012).

#### Superfamily Opomyzoidea (O Lonsdale)

JF McAlpine’s (1989) superfamily Opomyzoidea is largely derived from Hennig’s (1971) concept of Anthomyzoidea, but it is a highly problematic and likely polyphyletic entity that remains in use mostly as a convenient grouping for numerous families and genera of uncertain placement. Winkler et al. (2010) used molecular data to show that the superfamily is non-monophyletic. The boundaries of Aulacigastridae and Periscelididae have undergone considerable permutation, resulting in some stability and recognition of the new family Neminidae (discussion in Rung and Mathis (2011)), but placement and status of the genera allied to *Stenomicra* Coquillet are still uncertain (Winkler et al. 2010, Marshall 2012).

With 450 species, the Agromyzidae are by far the most diverse opomyzoid family in Canada. More than a hundred species have been added since 1979 but the large number of BINs (772) (Table 1) suggest that much taxonomic work remains to be done, especially in the large genus *Phytomyza* Fallén, which contains many undescribed species. The remaining families are relatively species-poor and often uncommon in Canada. These consist of the Asteiidae (5 species in Canada), Aulacigastridae (2), Periscelididae (3), Odiniidae (6), Opomyzidae (11), Anthomyzidae (37) and Clusiidae (22). Numbers of Canadian species for these small families have remained relatively constant since 1979 with the exception of the Anthomyzidae, which increased nine-fold through the revisionary work of Roháček and Barber (2016), and the Clusiidae, which were fully revised over the past 20 years (e.g., Lonsdale et al. 2011, Lonsdale 2017b; Table 1). A few additional Canadian species are expected for each family except the Clusiidae (Table 1).

The Canadian Agromyzidae were revised by Spencer (1969), but since that time generic concepts have been extensively reconsidered, especially in Winkler et al. (2009) and Lonsdale (2014), and several genera have been revised: *Amauromyza* Hendel (Boucher 2012b); *Cerodontha* Rondani (Boucher 2002, 2003, 2008, 2012a); *Liriomyza* Mik (Lonsdale 2017a); *Pseudonapomyza* Hendel (Boucher 2004). Many north temperate species of *Phytomyza* (sometimes as “*Chromatomyia* Hardy”) were treated in a long series of papers by GCD Griffiths. Apart for the Anthomyzidae and the Clusiidae (see above), little has been published about the remaining families since 1979. Rung and Mathis (2011) globally revised *Aulacigaster* Macquart (the only Aulacigastridae occurring in Canada) and a new invasive Opomyzidae was recorded by Wheeler et al. (1999).

Winkler et al. (2009) summarized known biologies, which often includes phytophagy with a number of Agromyzidae being highly pestiferous, but there are also associations with fungi, sap fluxes, frass, and insect galleries in trees. Some taxa are predaceous and a few have larvae that are aquatic to semi-aquatic; Rotheray and Horsfield (2013) found Clusiidae to feed on biofilm in decaying wood.

#### Superfamily Carnoidea (JF Gibson)

The families currently in Carnoidea (Marshall 2012) were divided amongst the superfamilies Anthomyzoidea and Drosophiloidea in JF McAlpine et al. (1979). Another recent change to the classification of the group involves the inclusion of the family formerly known as the Tethinidae in the Canacidae (DK McAlpine 2007a). JF McAlpine (1989) determined “Carnites” Newman (1834) as the oldest family-level name in the group, thus making Carnoidea the proper superfamily name rather than Chloropoidea.

Of the five Carnoidea families in Canada (Table 1), three have few species and only minor changes have occurred since 1979: Acartophthalmidae (1 species in Canada), Carnidae (12), and Milichiidae (13). Canacidae were globally catalogued recently (Munari and Mathis 2010) and the number of reported Canadian species has doubled since 1979 to a current total of 10. More carnids and milichiids remain to be discovered in Canada and species numbers are expected to at least double for these families (Table 1). Much recent work has been completed on Chloropidae, both globally (e.g., Nartshuk 2012) and within Canada only (e.g., Grégoire Taillefer and Wheeler 2011, Barrie and Wheeler 2016). These publications have resulted in 40 more species of Chloropidae reported here compared to JF McAlpine et al. (1979), but many more species of this family are suspected to be undescribed or unreported based on BIN numbers and field observations. Members of Carnoidea display a wide range of life histories including saprophagous larvae, coprophagous larvae, kleptoparasitism (Milichiidae), crop pests (some Chloropidae), and parasites in bird’s nests (Carnidae).

#### Superfamily Ephydroidea (JF Gibson)

The families currently in Ephydroidea (Marshall 2012) were divided amongst the superfamilies Anthomyzoidea and Drosophiloidea in JF McAlpine et al. (1979). The present configuration reflects the most recent phylogenetic hypotheses and correctly identifies Ephydridae as the oldest valid family-group name in the taxon (JF McAlpine 1989, Grimaldi 1990). The recent molecular analysis of Wiegmann et al. (2011) supported a monophyletic Ephydroidea.

Of the six families in Canada (Table 1), four have few species recorded from the country and have not seen a marked change in species numbers since JF McAlpine et al. (1979): Braulidae (1 species in Canada), Diastatidae (7), Curtonotidae (1), and Camillidae (2). A few additional species of Diastatidae are expected (Table 1). Both Drosophilidae and Ephydridae have seen numbers of species reported in Canada increase by approximately 30% since JF McAlpine et al. (1979), to 79 and 197 species, respectively, and more unreported and undescribed species are likely to be found (Table 1). Nearctic Drosophilidae have been the subject of considerable phylogenetic, taxonomic, and faunistic research (e.g., Remsen and O’Grady 2002, Brake and Bächli 2008, Miller et al. 2017) and many of the genera and subgroups of Nearctic Ephydridae have been revised recently (e.g., Mathis and Zatwarnicki 1995, Costa et al. 2016). Most species of Ephydroidea are suspected to be saprophagous as larvae, although there have been records of leaf-mining and predaceous species. Some species of Ephydridae are noteworthy as extremophiles, including hot spring, salt water, and petroleum-inhabiting species. Some Drosophilidae are important model species in genetic research and the family also includes a number of agricultural pests.

#### Superfamily Sphaeroceroidea (SA Marshall and O Lonsdale)

In Canada, this superfamily contains three families (Table 1). While detailed study is required, consensus thus far is that the family Heleomyzidae is rendered paraphyletic by the Sphaeroceridae (Roháček et al. 2001). While some authors have suggested that Sphaeroceridae and Heleomyzidae should be combined (DK McAlpine 2007b), others have suggested dividing the Heleomyzidae into multiple families, as discussed in JF McAlpine (1989), DK McAlpine (1985, 2007b), and Papp (1998). It appears most likely that the Heleomyzidae will be broken up once the phylogeny of the group is better resolved.

The infrequently encountered Chyromyidae include five recorded species in Canada with perhaps as many more awaiting discovery (Table 1). The more heterogeneous Heleomyzidae currently have 72 recorded species in Canada, including four species previously treated as Trixoscelidae by JF McAlpine et al. (1979), and as many as 38 additional species are expected (Table 1). Most species in the superfamily belong to the well-defined family Sphaeroceridae, which include thousands of species worldwide (catalogued by Roháček et al. 2001). The Canadian Sphaeroceridae have been fully revised since 1979, resulting in a five-fold increase in species numbers (from 35 to 184); relatively few additions are expected as further collecting is carried out (Table 1).

Canadian Sphaeroceridae can be identified to genus using the keys in Marshall and Richards (1987) and in Marshall and Buck (2010); almost all Canadian species can be identified with keys cited in the latter work. The Canadian Chyromyidae can be keyed using Malloch (1914) and Wheeler (1961), but the fauna should be re-examined as it is probable that undescribed species remain to be discovered. The Canadian Heleomyzidae were remarkably well covered by Gill (1962, 1965); there have been few changes since then but new synonymies and additional taxa are to be expected, especially once the genus *Suillia* Robineau-Desvoidy is revised.

Sphaeroceridae develop as microbial grazers in a wide variety of moist microhabitats, including dung, carrion, fungi and many kinds of decaying plant material. Many inhabit mammal nests or burrows, and several species are associated with caves. Heleomyzidae have similar habits and also occur in caves, mammal nests, bird’s nests, fungi, and dung. Some Chyromyidae have also been reared from bird’s nests. Immature stages of Sphaeroceroidea are poorly known with the exception of the specialized coastal species found in decomposing seaweed (Marshall 1982).

### Schizophora: Calyptratae

This large monophyletic subgroup of Schizophora has received much systematic attention over the last three decades (e.g., McAlpine 1989, Nirmala et al. 2001, Kutty et al. 2008, 2010, Zhang et al. 2016). Whereby JF McAlpine et al. (1979) grouped all calyptrates into a single superfamily (Muscoidea), most recent published works (Kutty et al. 2010, Wiegmann et al. 2011, Lambkin et al. 2013, Cerretti et al. 2017) have supported a division of the group into the Hippoboscoidea, the paraphyletic ‘muscoid grade’, and the Oestroidea (nested in the muscoid grade) proposed by Kutty et al. (2008). The composition of the Calyptratae has remained mostly unchanged since Roback (1951), and ten of the approximately 15 recognized calyptrate families worldwide (Cerretti et al. 2017) occur in Canada.

#### Superfamily Hippoboscoidea (J Savage)

The Hippoboscoidea are presently considered the sister-group to the remaining calyptrates (Kutty et al. 2008, 2010). It includes the Glossinidae (tsetse flies) and the Hippoboscidae (louse flies and batflies) with only the latter family found in Canada. JF McAlpine et al. (1979) recognized the families Nycteribiidae and Streblidae, which are now included as subfamilies of the Hippoboscidae (Kutty et al. 2010, Pape et al. 2011). The Canadian batflies, represented by two species of wingless spider-like nycteribiines and one small hairy strebline are only known from western provinces (British Columbia and Saskatchewan) (Wenzel 1965, Graciolli et al. 2007). With the exception of the Nycteribiinae (Graciolli et al. 2007), there has been little work done on the Canadian Hippoboscidae fauna since 1979. While 17 species are currently recorded from Canada, DNA barcodes from Canadian hippoboscid specimens are few and all placed in a single BIN, further emphasizing the need for additional field collecting and taxonomic work on this group.

All Hippoboscidae are larviparous and deposit mature larvae that are ready to pupate. The adults have a striking appearance that reflects their ectoparasitic habits and many species have limited or no flying abilities. The stocky, dorsoventrally flattened Hippoboscinae will feed on the blood of many birds and mammal species while the Streblinae and Nycteribiinae are restricted to bats.

## “Muscoid Grade” (J Savage)

While we acknowledge the paraphyly of the muscoid grade, the group is used here for convenience as no alternative classification scheme has yet been proposed to assign the muscoid families to higher taxa. Members of this assemblage can be recognized mostly by the absence of diagnostic features found in the Hippoboscoidea (e.g., adaptations to ectoparasitic habits) and the Oestroidea (e.g., meron with a row of strong setae). The most important change relating to the Canadian fauna since JF McAlpine et al. (1979) is the recognition of the Fanniidae as distinct from the Muscidae (Griffiths 1972, McAlpine 1989)

All four muscoid families are found in Canada. In his census of Canadian Diptera, JF McAlpine et al. (1979) reported more muscid than anthomyiid species (525 vs 375) but the subsequent recognition of the Fanniidae as a separate family (84 species in Canada) and numerous synonymies have reduced the number of Canadian muscids to 440. The publication of more than a hundred new anthomyiid species and records (Griffiths 1982–2004) have resulted in a total of 515 documented species of Anthomyiidae and the group has supplanted the muscids as the most species-rich muscoid family in Canada. The Scathophagidae currently have 126 recorded species in Canada but have received less taxonomic attention than the other taxa. BINs are close to the numbers of described species for all families in this group and an increase in species numbers of only 10–20% is expected in the future (Table 1).

The Anthomyiidae are the only muscoid family to have been recently revised for Canada (Griffiths 1982–2004, excluding *Botanophila* Lioy and *Fucellia* Robineau-Desvoidy). A few generic and type revisions (e.g., Cuny 1980, Pont 1984, 2011, Savage 2003, Moores and Savage 2005) as well as some faunistic contributions (e.g., Renaud et al. 2012a, b) have nonetheless improved our knowledge of the taxonomy and distribution of Canadian Muscidae and Fanniidae. The Scathophagidae, unfortunately, have remained mostly unstudied.

In Canada, muscoid flies are especially well represented in northern and alpine habitats (Huckett 1965, Griffiths 1982–2004). The saprophagous housefly (*Muscadomestica* Linnaeus) is the best known member of the group, but immature and adult muscoids exhibit a range of ecological habits so broad that it almost spans the complete spectrum displayed at the order level (see Marshall (2012) and Courtney et al. (2017) for general overview).

### Superfamily Oestroidea (JE O’Hara)

This large lineage of nearly 15,000 species worldwide (Pape et al. 2011) has long been recognized as monophyletic based on morphology (Griffiths 1972, McAlpine 1989) and this view has since been corroborated by molecular analyses (Kutty et al. 2010, Wiegmann et al. 2011, Marinho et al. 2012). Some major family-level changes have been recently implemented in the Oestroidea (see Ceretti et al. 2017), but the same five families recognized by JF McAlpine et al. (1979) are still recognized today (Table 1).

With 736 known species, the Tachinidae have a large presence in Canada (O’Hara and Wood 2004, J O'Hara and M Wood unpubl. data), placing them second behind the Chironomidae as the most speciose family of Diptera in the country based on numbers of described species (Table 1). More than 200 species have been added to the Canadian fauna since 1979, mostly as a result of tribal (Wood 1985, O’Hara 1989, 2002) and generic revisions (e.g., O’Hara 1983, 1994, 2012, Sun and Marshall 2003). Despite these advances, dozens of undescribed Canadian species are still awaiting description in collections. Much recent attention has also been dedicated to the Sarcophagidae (135 species in Canada) and the Calliphoridae (62) and their species numbers have increased by over 50% since 1979 (Table 1). Catalogues and revisions that account for most of the increase in Canadian numbers of sarcophagid species include Pape (1996), Dahlem and Naczi (2006), and Giroux and Wheeler (2009, 2010). A few additional species of sarcophagids are expected in the country. The Canadian calliphorid fauna is very well known and recent taxonomic tools to identify it include Sabrosky et al. (1989), Rognes (1991), Whitworth (2006), Marshall et al. (2011), Jewiss-Gaines et al. (2012), and Tantawi et al. (2017). No additional species are expected in Canada. The Oestridae (17 species in Canada) and introduced Rhinophoridae (2) have not undergone any significant changes since JF McAlpine et al. (1979) (Table 1). The correspondence between BINs and known species numbers is generally good in this superfamily except for the Oestridae and, to a lesser extent, the Calliphoridae (Table 1).

The Oestroidea are generally large robust flies that display a wide range of life histories and ecological habits. The Tachinidae and Rhinophoridae are all parasitoids of terrestrial arthropods and the larvae of Oestridae are internal parasites of wild or domestic mammals. The calliphorids include a few parasitoid species in the *Pollenia* Robineau-Desvoidy complex, all presumably introduced from Europe with their earthworm hosts (Rognes 1991), and some ectoparasites of birds (*Protocalliphora* Hough) but most are saprophagous and associated with decaying animal matter. The Sarcophagidae have the most diverse larval habits of the superfamily; some are kleptoparasites in the nests of solitary bees and wasps, some feed on carrion or dung, and others are associated with living animals as parasitoids (particularly arthropods) or predators. Several parasitic species of Oestridae, Sarcophagidae and Calliphoridae are also known to cause myiasis in humans and other vertebrates (Marshall 2012).

## References

[B1] Adler PH Crosskey RW (2018) World blackflies (Diptera: Simuliidae: a comprehensive revision of the taxonomic and geographical inventory. https://biomia.sites.clemson.edu/pdfs/blackflyinventory.pdf [accessed IV.2018]

[B2] Adler PH Currie DC Wood DM (2004) The Black flies (Simuliidae) of North America. Cornell University Press, Ithaca, New York, 941 pp.

[B3] Amorim DS (1993) A phylogenetic analysis of the basal groups of Bibionomorpha, with a critical reanalysis of the wing vein homology. Revista Brasileira de Biologia 52: 379–399.

[B4] Amorim DS Rindal E (2007) Phylogeny of the Mycetophiliformia, with proposal of the subfamilies Heterotrichinae, Ohakuneinae, and Chiletrichinae for the Rangomaramidae (Diptera, Bibionomorpha). Zootaxa 1535: 1–92.

[B5] Andersen T Cranston PS Epler JH (2013) Chironomidae of the Holarctic Region. Keys and diagnoses – larvae. Insect Systematics & Evolution Supplement 66: 1–573.

[B6] Arnaud PH Jr Boussy IA (1994) The adult Thaumaleidae (Diptera: Culicomorpha) of Western North America. Myia 5: 41–152.

[B7] Ashe P O’Connor JP (2009) A World Catalogue of Chironomidae (Diptera). Part 1. Buchonomyiinae, Chilenomyiinae, Podonominae, Aphroteniinae, Tanypodinae, Usambaromyiinae, Diamesinae, Prodiamesinae and Telmatogetoninae. Irish Biogeographical Society and National Museum of Ireland, Dublin, 445 pp.

[B8] Ashe P O’Connor JP (2012) A World Catalogue of Chironomidae (Diptera) Part 2, Orthocladiinae A and B. Irish Biogeographical Society and National Museum of Ireland, Dublin, 968 pp.

[B9] Bächli G (2018) Taxodros: A taxonomic database of the Drosophilidae [online]. https://www.taxodros.uzh.ch/ [accessed II.2018]

[B10] Barber KN (1985) A revision of *Pseudodinia* Coquillet (Diptera: Chamaemyiidae). Proceedings of the Entomological Society of Ontario 116: 105–167.

[B11] Barber KN (2006) *Strongylophthalmyiapengellyi* n. sp., a second species of Nearctic Strongylophthalmyiidae (Diptera). Journal of the Entomological Society of Ontario 137: 81–109.

[B12] Barrie CL Wheeler TA (2016) Revision of the Nearctic species of *Dicraeus* Loew (Diptera: Chloropidae). The Canadian Entomologist 148: 375–395. 10.4039/tce.2015.74

[B13] Bechev D Chandler P (2011) Catalogue of the Bolitophilidae and Diadocidiidae of the World (Insecta: Diptera). Zootaxa 2741: 38–58.

[B14] Bertone MA Courtney GW Wiegmann BM (2008) Phylogenetics and temporal diversification of the earliest true flies (Insecta: Diptera) based on multiple nuclear genes. Systematic Entomology 33: 668–687. 10.1111/j.1365-3113.2008.00437.x

[B15] Borkent A (1979) Systematics and bionomics of the species of the subgenus Schadonophasma Dyar and Shannon (*Chaoborus*, Chaoboridae, Diptera). Quaestiones Entomologicae 15: 122–255.

[B16] Borkent A (1981) The distribution and habitat preferences of the Chaoboridae (Culicomorpha: Diptera) of the Holarctic Region. Canadian Journal of Zoology 59: 122–133. 10.1139/z81-019

[B17] Borkent A (2008) The frog-biting midges of the world (Corethrellidae: Diptera). Zootaxa 1804: 1–456.

[B18] Borkent A (2012) The pupae of Culicomorpha – morphology and a new phylogenetic tree. Zootaxa 3396: 1–98.

[B19] Borkent A (2014) The pupae of the Biting Midges of the World (Diptera: Ceratopogonidae), with a generic key and analysis of the phylogenetic relationships between genera. Zootaxa 3879: 1–327. 10.11646/zootaxa.3879.1.1 25544570

[B20] Borkent A (2016) World Species of Biting Midges (Diptera: Ceratopogonidae). http://www.inhs.illinois.edu/research/FLYTREE/Borkent.html [accessed 9.III.2018]

[B21] Borkent A (2018) The state of phylogenetic analysis: narrow visions and simple answers —examples from the Diptera (flies). Zootaxa 4374: 107–143. 10.11646/zootaxa.4374.1.7 29689817

[B22] Borkent A Brown BV Adler PH Amorim DS Barber K Bickel D Boucher S Brooks SE Burger J Burington ZL Capellari RS Costa DNR Cumming JM Curler G Dick CW Epler JH Fisher E Gaimari SD Gelhaus J Grimaldi DA Hash J Hauser M Hippa H Ibáñez-Bernal S Jaschhof M Kameneva EP Kerr PH Korneyev V Korytkowski CA Kung G-A Kvifte GM Lonsdale O Marshall SA Mathis W Michelsen V Naglis S Norrbom AL Paiero S Pape T Pereira-Colavite A Pollet M Rochefort S Rung A Runyon JB Savage J Silva VC Sinclair BJ Skevington JH Stireman III JH Swann J Thompson FC Vilkamaa P Wheeler T Whitworth T Wong M Wood DM Woodley N Yau T Zavortink TJ Zumbado MA (2018) Remarkable fly (Diptera) diversity in a patch of Costa Rican cloud forest: Why inventory is a vital science. Zootaxa 4402: 53–90. 10.11646/zootaxa.4402.1.3 29690278

[B23] Borkent A Grogan W (2009) Catalog of the new world biting midges north of Mexico (Diptera: Ceratopogonidae). Zootaxa 2273: 1–48.

[B24] Borkent A Sinclair BJ (2012) The male genital tract of Axymyiidae and Tanyderidae (Diptera). The Canadian Entomologist 144: 266–272. 10.4039/tce.2012.26

[B25] Borkent CJ Gillung PJ Winterton SL (2016) Jewelled spider flies of North America: a revision and phylogeny of *Eulonchus* Gerstaecker (Diptera, Acroceridae). ZooKeys 169: 103–146. 10.3897/zookeys.619.8249 PMC5090163 27829790

[B26] Borkent CJ Wheeler TA (2012) Systematics and phylogeny of *Leptomorphus* (Diptera: Mycetophilidae). Zootaxa 3549: 1–117.

[B27] Boucher S (2002) Revision of Nearctic species of Cerodontha (Cerodontha) (Diptera: Agromyzidae). The Canadian Entomologist 134: 577–603. 10.4039/Ent134577-5

[B28] Boucher S (2003) The New World species of Cerodontha (Xenophytomyza) Frey (Diptera: Agromyzidae). Zootaxa 178: 1–8. 10.11646/zootaxa.178.1.1

[B29] Boucher S (2004) The New World fauna of the genus *Pseudonapomyza* (Diptera: Agromyzidae). The Canadian Entomologist 136: 781–791. 10.4039/n03-121

[B30] Boucher S (2008) A new species and taxonomic notes on northern Nearctic Cerodontha (Icteromyza) (Diptera: Agromyzidae. The Canadian Entomologist 140(5): 557–562. 10.4039/n08-033

[B31] Boucher S (2012a) Revision of the Nearctic species of Cerodontha (Icteromyza) (Diptera: Agromyzidae). The Canadian Entomologist 144: 122–157. 10.4039/tce.2012.12

[B32] Boucher S (2012b) Revision of the Canadian species of *Amauromyza* Hendel (Diptera: Agromyzidae). The Canadian Entomologist 144: 733–757. 10.4039/tce.2012.80

[B33] Brake I (2009) Revision of *Milichiella* Giglio-Tos (Diptera, Milichiidae). Zootaxa 2188: 1–166.

[B34] Brake I (2011) World catalog of the family Carnidae (Diptera, Schizophora). Myia 12: 113–169.

[B35] Brake I Bächli G (2008) World Catalogue of Insects: Volume 9 Drosophilidae (Diptera). Apollo Books, Copenhagen, 412 pp.

[B36] Brochu K Wheeler TA (2009) Systematics and ecology of Nearctic species of *Neophyllomyza* (Diptera: Milichiidae). The Canadian Entomologist 141(2): 103–111. 10.4039/n09-001

[B37] Brodo F (1987) A revision of the genus *Prionocera* (Diptera: Tipulidae). Evolutionary Monographs 8: 1–93.

[B38] Brodo F (1990) Crane flies (Diptera: Tipulidae) of the Arctic Islands. In: Harrington CR (Ed.) Canada’s missing dimension, Volume 2.Canadian Museum of Nature, Ottawa, 471–484.

[B39] Brodo F (2000) The insects, mites, and spiders of Hot Weather Creek, Ellesmere Island, Nunavut. Geological Survey of Canada Bulletin 529: 145–173. 10.4095/211956

[B40] Brooks SE Cumming JM (2017) Revision of the Nearctic *Parathalassius* Mik (Diptera: Dolichopodidae: Parathalassiinae), with a review of the world fauna. Zootaxa 4314: 1–64. 10.11646/zootaxa.4314.1.1

[B41] Brown BV (1992) Generic revision of Phoridae of the Nearctic Region and phylogenetic classification of Phoridae, Sciadoceridae, and Ironomyiidae (Diptera). Memoirs of the Entomological Society of Canada 164: 1–144. 10.4039/entm124164fv

[B42] Brown BV (1995) Review of the species of *Anevrina* Lioy (Diptera: Phoridae), with a new species and a revised world key. Entomological Problems 25 [1994]: 1–10.

[B43] Brown BV Amorim DdS Kung G-A (2015) New morphological characters for classifying Phoridae (Diptera) from the structure of the thorax. Zoological Journal of the Linnaean Society 173: 424–485. 10.1111/zoj.12208

[B44] Brown BV Borkent AH Adler P Amorim DdS Barber K Bickel D Boucher S Brooks SE Burger J Burington ZL Capelari RS Costa DNR Cumming JM Curler G Dick CW Epler JH Fisher E Gaimari SD Gelhaus J Grimaldi DA Hash J Hauser M Hippa H Ibáñez-Bernal S Jaschhof M Kameneva EP Kerr PH Korneyev V Korytkowski CA Kung G-A Kvifte GM Lonsdale O Marshall SA Mathis W Michelsen V Naglis S Norrbom AL Paiero S Pape T Pereira-Colavite A Pollet M Rochefort S Rung A Runyon JB Savage J Silva VC Sinclair BJ Skevington JH Stireman III JO Swann J Thompson FC Vilkamaa P Wheeler T Whitworth T Wong M Wood DM Woodley N Yau T Zavortink TJ Zumbado MA (2018) Comprehensive inventory of true flies (Diptera) at a tropical site. Communications Biology 1: 1–8. 10.1038/s42003-018-0022-x 30271908 PMC6123690

[B45] Buck M Marshall SA (2006a) The identity of *Pseudopsila*, description of a new subgenus of *Psila*, and redefinition of *Psila* sensu lato (Diptera: Psilidae). European Journal of Entomology 103: 183–192. 10.14411/eje.2006.021

[B46] Buck M Marshall SA (2006b) Revision of New World *Loxocera* (Diptera: Psilidae), with phylogenetic redefinition of Holarctic subgenera and species groups. European Journal of Entomology 103: 193–219. 10.14411/eje.2006.021

[B47] Burger JF (1995) Catalog of Tabanidae (Diptera) of North America north of Mexico. Contributions on Entomology, International 1: 1–100.

[B48] Byers GW (1983) The crane fly genus *Chionea* in North America. The University of Kansas Science Bulletin 52: 59–195.

[B49] Byers GW (1992) Crane flies – three families or one. Acta Zoologica Cracoviensia 35: 37–41.

[B50] Caloren DC Marshall SA (1998) A revision of the genus *Clusiodes* (Clusiidae). Studia Dipterologica 5(2): 1–61.

[B51] Cannings RA (1994) Robber flies (Diptera: Asilidae) new to Canada, British Columbia, Yukon and the Northwest Territories with notes on distribution and habitat. Journal of the Entomological Society of British Columbia 103: 19–26.

[B52] Cannings RA (1997) Robber flies (Diptera: Asilidae) of the Yukon. In: Danks HV Downes JA (Eds) Insects of the Yukon.Biological Survey of Canada (Terrestrial Arthropods), Ottawa, 638–662.

[B53] Cannings RA (2002) The systematics of *Lasiopogon* (Diptera: Asilidae). Royal British Columbia Museum, Victoria, 354 pp.

[B54] Cannings RA (2006) A review of the distribution and natural history of *Apiocerabarri* and *Nemomydaspantherinus* (Diptera: Apioceridae and Mydidae), two rare asiloid flies from the southern Interior of British Columbia. Journal of the Entomological Society of British Columbia 103: 55–60.

[B55] Capelle KJ (1953) A revision of the genus *Loxocera* in North America, with a study of geographical variation in *L.cylindrical* (Diptera, Psilidae). Annals of the Entomological Society of America 46: 99–114. 10.1093/aesa/46.1.99

[B56] Cerretti P Stireman JO III Pape T O’Hara JE Marinho MAT Rognes K Grimaldi DA (2017) First fossil of an oestroid fly (Diptera: Calyptratae: Oestroidea) and the dating of oestroid divergences. PLoS ONE 12(8): e0182101. 10.1371/journal.pone.0182101 PMC5568141 28832610

[B57] Collins KP Wiegmann BM (2002) Phylogenetic relationships of the lower Cyclorrhapha (Diptera: Brachycera) based on 28S rDNA sequences. Insect Systematics and Evolution 33: 445–456. 10.1163/187631202X00235

[B58] Cook EF (1981) Scatopsidae In: McAlpine JF, Peterson BV, Shewell GE, Teskey HJ, Vockeroth JR, Wood DM (Coordinators) Manual of Nearctic Diptera. Volume 1. Agriculture Canada Monograph 27, 313–324.

[B59] Costa DN Mathis WN Marinoni L (2016) A revision of the shore-fly genus *Lamproclasiopa* Hendel (Diptera, Ephydridae). ZooKeys 631: 1–99. 10.3897/zookeys.631.10718 PMC5126506 27917044

[B60] Courtney GW (1990) Revision of Nearctic mountain midges (Diptera: Deuterophlebiidae). Journal of Natural History 24: 81–118. 10.1111/j.1365-3113.1991.tb00683.x

[B61] Courtney GW (1994) Biosystematics of the Nymphomyiidae (Insecta: Diptera): life history, morphology, and phylogenetic relationships. Smithsonian Contributions to Zoology No. 550, iv + 41 pp.

[B62] Courtney GW (2000) Revision of the net-winged midges of the genus *Blepharicera* Macquart (Diptera: Blephariceridae) of eastern North America. Memoirs of the Entomological Society of Washington 23: 1–99.

[B63] Courtney GW Pape T Skevington JH Sinclair BJ (2017) Biodiversity of Diptera. In: Foottit RG Adler PH (Eds) Insect Biodiversity: Science and Society, Volume 1, 2nd Edition.Blackwell Publishing, Oxford, 229–278. 10.1002/9781118945568.ch9

[B64] Cumming HJ Wheeler TA (2016) Revision of the Nearctic species of *Callomyia* Meigen (Diptera: Platypezidae) and phylogeny of the genus. Zootaxa 4111: 501–554. 10.11646/zootaxa.4111.5.1 27395101

[B65] Cumming JM Sinclair BJ Brooks SE O’Hara JE Skevington JH (2011) The history of dipterology at the Canadian National Collection of Insects, with special reference to the Manual of Nearctic Diptera. The Canadian Entomologist 143(6): 539–577. 10.4039/n11-029

[B66] Cumming JM Sinclair BJ Wood DM (1995) Homology and phylogenetic implications of male genitalia in Diptera – Eremoneura. Entomologica Scandinavica 26: 121–151. 10.1163/187631295X00143

[B67] Cuny R (1980) Revision of the genus *Eudasyphora* Townsend (Diptera: Muscidae), and reflections on its evolution. The Canadian Entomologist 112(4): 345–373. 10.4039/Ent112345-4

[B68] Curry CJ Gibson JF Shokralla S Hajibabaei M Baird DJ (2018) Identifying North American freshwater invertebrates using DNA barcodes: are existing COI sequence libraries fit for purpose? Freshwater Science 37(1): 179–189. 10.1086/696613

[B69] Dahl C (1973) Trichoceridae (Dipt.) of Spitsbergen. In: Notes on the arthropod fauna of Spitsbergen III. Annals Entomologica Fennica 39: 49–59.

[B70] Dahlem GA Naczi RFC (2006) Flesh flies (Diptera: Sarcophagidae) associated with North American pitcher plants (Sarraceniaceae), with descriptions of three new species. Annals of the Entomological Society of America 99: 218–240. 10.1603/0013-8746(2006)099[0218:FFDSAW]2.0.CO;2

[B71] Danks HV (1979) Canada and its insect fauna. Memoirs of the Entomological Society of Canada No. 108, 373pp.

[B72] Darvas B Skuhravá M Andersen A (2000) Agricultural dipteran pests of the Palaearctic region. In: Papp L Darvas B (Eds) Contributions to a Manual of Palaearctic Diptera, Volume 1: General and Applied Dipterology.Science Herald, Budapest, 565–649

[B73] Disney RHL (1994) Continuing the debate relating to the phylogenetic reconstruction of the Phoridae (Diptera). Giornale Italiano di Entomologia 7: 103–117.

[B74] Disney RHL (2001) The preservation of small Diptera. Entomologists Monthly Magazine 137: 155–159.

[B75] Downes JA Wirth WW (1981) Ceratopogonidae In: McAlpine JF, Peterson BV, Shewell GE, Teskey HJ, Vockeroth JR, Wood DM (Coordinators) Manual of Nearctic Diptera, Vol 1. Agriculture Canada Monograph 27, 393–421.

[B76] Evenhuis NL (2002) Catalog of the Mythicomyiidae of the World (Insecta: Diptera). Bishop Museum Bulletin in Entomology 10: 1–85.

[B77] Evenhuis NL (2006) Catalog of Keroplatidae of the world. Bishop Museum Bulletin in Entomology 13: 1–178.

[B78] Evenhuis NL Greathead DJ (1999) World catalog of bee flies (Diptera: Bombyliidae). Backhuys Publishers, Leiden, 756 pp.

[B79] Fasbender A Courtney GW (2017) A revision of Bittacomorphinae with a review of the monophyly of extant subfamilies of Ptychopteridae (Diptera). Zootaxa 4309: 1–69. 10.11646/zootaxa.4309.1.1

[B80] Feijen HR (1989) Cyclorrhapha 3 (Schizophora other than Calyptratae). Diopsidae. Flies of the Nearctic Region 9: 1–122.

[B81] Fitzgerald SJ Kerr PH (2014) Revision of Nearctic *Phthinia* Winnertz (Diptera: Mycetophilidae). Zootaxa 3856: 301–325. 10.11646/zootaxa.3856.3.1 25284661

[B82] Fitzgerald SJ Wood DM (2014) A new species of Axymyiidae (Diptera) from western North America and a key to the Nearctic species. Zootaxa 3857: 101–113. 10.11646/zootaxa.3857.1.4 25283098

[B83] Foote RH Blanc FL Norrbom AL (1994) Handbook of the Fruit Flies (Diptera: Tephritidae) of America North of Mexico. Comstock Publishing Associates, Ithaca, New York, xii + 571 pp.

[B84] Friedrich M Tautz D (1997) Evolution and phylogeny of the Diptera: a molecular phylogenetic analysis using 28S rDNA sequences. Systematic Biology 46: 674–698. 10.1093/sysbio/46.4.674 11975338

[B85] Gaimari S Mathis W (2011) World Catalog and Conspectus on the Family Odiniidae (Diptera: Schizophora). Myia 12: 291–339.

[B86] Gagné RJ (1981) A monograph of *Trichonta* with a model for the distribution of Holarctic Mycetophilidae (Diptera). United States Department of Agriculture Technical Bulletin No. 1638, 64 pp.

[B87] Gagné RJ (1989) The Plant Feeding Gall Midges of North America. Cornell University Press, Ithaca, New York, xi + 356 pp.

[B88] Gagné RJ (2018) Key to adults of North American genera of the subfamily Cecidomyiinae (Diptera: Cecidomyiidae). Zootaxa 4392: 401–457. 10.11646/zootaxa.4392.3.1 29690392

[B89] Gagné RJ Jaschhof M (2017) A Catalog of the Cecidomyiidae (Diptera) of the World, 4^th^ Edition. Digital. 762 pp. https://www.ars.usda.gov/ARSUserFiles/80420580/Gagne_2017_World_Cat_4th_ed.pdf [accessed 18.VI.2018]

[B90] Gathmann FO Williams DD (2006) Insect emergence in Canadian coldwater springs: spatial and temporal patterns, and species-environment relationships. Annales de Limnologie 42: 143–156. 10.1051/limn/2006015

[B91] Gelhaus JK (2001) European Crane Flies *Tipula* spp. In: FY2002 Eastern Region Cooperative Agriculture Pest Survey (CAPS) Guidelines. National Plant Board, 13–17.

[B92] Gelhaus JK (2005) Systematics and biogeography of the desert crane fly subgenus Tipula (Eremotipula) Alexander (Diptera: Tipulidae). Memoirs of the American Entomological Society 46: 1–235.

[B93] Giordano BV Gasparotto A Hunter FF (2015) A checklist of the 67 mosquito species of Ontario, Canada. Journal of the American Mosquito Control Association 31(1): 101–103. 10.2987/14-6456R.1 25843183

[B94] Gibson JF (2017) An updated and annotated checklist of the thick-headed flies (Diptera: Conopidae) of British Columbia, the Yukon, and Alaska. Journal of the Entomological Society of British Columbia 114: 38–55.

[B95] Gibson JF Skevington JH (2013) Phylogeny and taxonomic revision of all genera of Conopidae (Diptera) based on morphological data. Zoological Journal of the Linnean Society 167: 43–81. 10.1111/j.1096-3642.2012.00873.x 34818824

[B96] Gill GD (1962) The heleomyzid flies of America north of Mexico (Diptera: Heleomyzidae). Proceedings of the United States National Museum 113: 495–603. 10.5479/si.00963801.113-3465.495

[B97] Gill GD (1965) Family Heleomyzidae. In: Stone A Sabrosky CW Wirth WW Foote RH Coulson JR (Eds) A catalog of the Diptera of America north of Mexico.United States Department of Agriculture, Agriculture Handbook No. 276, 808–816.

[B98] Giroux M Wheeler TA (2009) Systematics and phylogeny of the subgenus Sarcophaga (Neobellieria) (Diptera: Sarcophagidae). Annals of the Entomological Society of America 102: 567–587. 10.1603/008.102.0401

[B99] Giroux M Wheeler TA (2010) Systematics of *Bulbostyla*, a new subgenus of *Sarcophaga* Meigen, and change of status for *Robackina* Lopes (Diptera: Sarcophagidae). Zootaxa 2553: 35–59.

[B100] Graciolli G Autino A Claps G (2007) Catalogue of American Nycteribiidae. Revista Brasileira de Entomologia 51(2): 142–159. 10.1590/S0085-56262007000200004

[B101] Grégoire Taillefer A Wheeler TA (2011) The genus *Calamoncosis* in the Nearctic region (Diptera: Chloropidae). The Canadian Entomologist 143: 652–661. 10.4039/n11-040

[B102] Greenwalt DE Moulton JE (2016) The first fossil New World Dixidae with a critical discussion of generic definitions. Palaeontologia Electronica 19.3.55A: 1–32. 10.26879/656

[B103] Grichanov IY Brooks SE (2017) Dolichopodidae (dolichopodid dance flies). In: Kirk-Spriggs AH Sinclair BJ (Eds) Manual of Afrotropical Diptera.Volume 2. Nematocerous Diptera and lower Brachycera. Suricata 5, South African National Biodiversity Institute, Pretoria, 1265–1320.

[B104] Griffiths GCD (1972) The phylogenetic classification of DipteraCyclorrhapha, with special reference to the structure of the male postabdomen. Series Entomologica 8, 340 pp.

[B105] Griffiths GCD (1982–2004) Anthomyiidae. In: Griffiths GCD (Ed.) Flies of the Nearctic Region 8(2): 1–2635.

[B106] Grimaldi DA (1990) A phylogenetic, revised classification of genera in the Drosophilidae (Diptera). Bulletin of the American Museum of Natural History 197: 1–139.

[B107] Grimaldi D Engel MS (2005) Evolution of the Insects. Cambridge University Press, New York, 755 pp.

[B108] Hall JC (1981) Bombyliidae In: McAlpine JF, Peterson BV, Shewell GE, Teskey HJ, Vockeroth JR, Wood DM (Coordinators) Manual of Nearctic Diptera. Volume 1. Agriculture Canada Monograph 27, 589–602.

[B109] Han HY Ro KE (2016) Molecular phylogeny of the superfamily Tephritoidea (Insecta: Diptera) reanalysed based on expanded taxon sampling and sequence data. Journal of Zoological Systematics and Evolutionary Research 54(4): 276–288. 10.1111/jzs.12139

[B110] Hebert PDN Cywinska A Ball SL deWaard JR (2003) Biological identification through DNA barcodes. Proceedings of the Royal Society of London B 270: 313–321. 10.1098/rspb.2002.2218 PMC1691236 12614582

[B111] Hebert PDN Ratnasingham S Zakharov EV Telfer AC Levesque–Beaudin V Milton MA Pedersen S Jannetta P de Waard JR (2016) Counting animal species with DNA barcodes: Canadian insects. Philosophical Transactions of the Royal Society B 371: 20150333. 10.1098/rstb.2015.0333 PMC4971185 27481785

[B112] Hennig W (1958) Die Familien der DipteraSchizophora und ihre phylogenetischen Verwandtschaftsbeziehungen. Beiträge zur Entomologie 8: 505–688.

[B113] Hennig W (1965) Die Acalyptratae des Baltischen Bernsteins und ihre Bedeutung fur die Erforschung der phylogenetischen Entwicklung dieser Dipteren-Gruppe. Stuttgarter Beiträge zur Naturkunde 145: 1–215.

[B114] Hennig W (1971) Neue Untersuchungen uber die Familien der DipteraSchizophora (Diptera: Cyclorrhapha). Stuttgarter Beiträge zur Naturkunde 226: 1–76.

[B115] Hennig W (1973) Diptera (Zweiflügler). Handbuch der Zoologie 4 (2) 2/31: 1–337.

[B116] Hoebeke ER Wheeler AG (1994) *Micropezacorrigiolata* (L.), a Eurasian stilt-legged fly (Diptera: Micropezidae) new to North America: Redescription, geographic distribution, and bionomics. Proceedings of the Entomological Society of Washington 96(3): 46–70.

[B117] Hogue CL (1987) Blephariceridae. In: Griffiths GCD (Ed.) Flies of the Nearctic Region 2(4): 1–172.

[B118] Huckett HC (1965) The Muscidae of Northern Canada, Alaska and Greenland (Diptera). Memoirs of the Entomological Society of Canada 42: 1–369. 10.4039/entm9742fv

[B119] Irwin ME Lyneborg L (1981) Therevidae In: McAlpine JF, Peterson BV, Shewell GE, Teskey HJ, Vockeroth JR, Wood DM (Coordinators) Manual of Nearctic Diptera, Volume 1. Agriculture Canada Monograph 27, 513–523.

[B120] Jackson M Howay T Belton P (2013) The first record of *Culisetaparticeps* (Diptera: Culicidae) in Canada. The Canadian Entomologist 145: 115–116. 10.4039/tce.2012.99

[B121] Jackson MD Marshall SA Hanner R Norrbom AL (2011) The Fruit Flies (Tephritidae) of Ontario. Canadian Journal of Arthropod Identification 15: 1–251. 10.3752/cjai.2011.15

[B122] Jaschhof M Jaschhof C (2009) The wood midges (Diptera: Cecidomyiidae: Lestremiinae) of Fennoscandia and Denmark. Studia Dipterologica 18 (Supplement): 1–333. 10.4289/0013-8797-111.4.909

[B123] Jaschhof M Jaschhof C (2013) The Porricondylinae (Diptera: Cecidomyiidae) of Sweden, with notes on extralimital species. Studia Dipterologica 20 (Supplement): 1–392.

[B124] Jewiss-Gaines A Marshall SA Whitworth TL (2012) Cluster flies (Calliphoridae: Polleniinae: *Pollenia*) of North America. Canadian Journal of Arthropod Identification 19: 1–22. 10.3752/cjai.2012.19

[B125] Kerr PH (2010) Phylogeny and classification of Rhagionidae, with implications for Tabanomorpha (Diptera: Brachycera). Zootaxa 2592: 1–133.

[B126] Kerr PH (2011) Six new species of *Acomoptera* from North America (Diptera, Mycetophilidae). ZooKeys 137: 41–76. 10.3897/zookeys.137.1764 PMC3260888 22259304

[B127] Kessel EL Buegler ME (1972) A review of the genus *Callomyia* in North America, with the description of a new species (Diptera: Platypezidae). The Wasmann Journal of Biology 30: 241–278.

[B128] Kevan DK McE Cutten FEA (1981) Nymphomyiidae In: McAlpine JF, Peterson BV, Shewell GE, Teskey HJ, Vockeroth JR, Wood DM (Coordinators) Manual of Nearctic Diptera, Volume 1. Agriculture Canada Monograph 27, 203–207.

[B129] Kits JH Marshall SA Evenhuis NL (2008) The bee flies (Diptera: Bombyliidae) of Ontario, with a key to the species of eastern Canada. Canadian Journal of Arthropod Identification 6. 10.3752/cjai.2008.06

[B130] Klymko J Marshall SA (2008) Review of the Nearctic Lonchopteridae (Diptera), including descriptions of three new species. The Canadian Entomologist 140: 649–673. 10.4039/n08-034

[B131] Klymko J Marshall SA (2011) Systematics of New World *Curtonotum* Macquart (Diptera: Curtonotidae). Zootaxa 3079: 1–110.

[B132] Knab F Shannon RC (1916) Tanypezidae in the United States of America (DipteraAcalyptrata). Insecutor Inscitiae Menstruus 4: 33–36.

[B133] Knutson L Orth RE Fisher TW Murphy WL (1986) Catalog of Sciomyzidae of America North of Mexico. Entomography 4: 1–53.

[B134] Knutson LV Vala J-C (2011) Biology of Snail-Killing Flies. Cambridge University Press, Cambridge, 584 pp.

[B135] Koenig DP Young CW (2007) First observation of parasitic relations between big-headed flies, *Nephrocerus* Zetterstedt (Diptera: Pipunculidae) and crane flies, *Tipula* Linnaeus (Diptera: Tipulidae: Tipulinae), with larval and puparial descriptions for the genus *Nephrocerus*. Proceedings of the Entomological Society of Washington 109: 52–65.

[B136] Korneyev VA (2000a) Phylogenetic relationships among the families of the superfamily Tephritoidea. In: Aluja M Norrbom AL (Eds) Fruit Flies (Tephritidae): Phylogeny and Evolution of Behavior.CRC Press, Boca Raton, Florida, 3–22.

[B137] Korneyev VA (2000b) Phylogenetic relationships among higher groups of Tephritidae. Phylogenetic relationships among the families of the superfamily Tephritoidea. In: Aluja M Norrbom AL (Eds) Fruit Flies (Tephritidae): Phylogeny and Evolution of Behavior.CRC Press, Boca Raton, Florida, 73–113.

[B138] Kutty SN Pape T Pont A Wiegmann BM Meier R (2008) The Muscoidea (Diptera: Calyptratae) are paraphyletic: Evidence from four mitochondrial and four nuclear genes. Molecular Phylogenetics and Evolution 49(2): 639–652. 10.1016/j.ympev.2008.08.012 18793735

[B139] Kutty SN Pape T Wiegmann BM Meier R (2010) Molecular phylogeny of the Calyptratae (Diptera: Cyclorrhapha) with an emphasis on the superfamily Oestroidea and the position of Mystacinobiidae and McAlpine’s fly. Systematic Entomology 35(4): 614–635. 10.1111/j.1365-3113.2010.00536.x

[B140] Kutty SN Wong WH Meusemann K Meier R Cranston PS (2018) A phylogenomic analysis of Culicomorpha (Diptera) resolves the relationships among the eight constituent families. Systematic Entomology 43(3): 434–446. 10.1111/syen.12285

[B141] Lambkin CL Sinclair BJ Pape T Courtney GW Skevington JH Meier R Yeates DK Blagoderov V Wiegmann BM (2013) The phylogenetic relationships among infraorders and superfamilies of Diptera based on morphological evidence. Systematic Entomology 38(1): 164–179. 10.1111/j.1365-3113.2012.00652.x

[B142] Langor DW (2019) The diversity of terrestrial arthropods in Canada. In: Langor DW Sheffield CS (Eds) The Biota of Canada – A Biodiversity Assessment. Part 1: The Terrestrial Arthropods.ZooKeys 819: 9–40. 10.3897/zookeys.819.31947 PMC6355749 30713431

[B143] Lefebvre V Fontaine C Villemant C Daugeron C (2014) Are empidine dance flies major flower visitors in alpine environments? A case study in the Alps, France. Biology Letters 10: 0140742. 10.1098/rsbl.2014.0742 PMC4261866 25376804

[B144] LeSage L Harrison AD (1981) Observations on the diversity, flight periods, emergence, swarming, and microdistribution of crane-flies at Salem Creek, Ontario (Diptera, Tipulidae, Ptychopteridae, and Trichoceridae). Aquatic Insects 3: 81–97. 10.1080/01650428109361051

[B145] Lonsdale O (2013) Review of the families Tanypezidae and Strongylophthalmyiidae, with a revision of *Neotanypeza* Hendel (Diptera: Schizophora). Smithsonian Contributions to Zoology No. 641, 60 pp.

[B146] Lonsdale O (2014) Redefinition and synonymy of genera in the *Ophiomyia* genus group, with the description of *Euhexomyza* new genus (Diptera: Agromyzidae). The Canadian Entomologist 146(5): 481–513. 10.4039/tce.2014.2

[B147] Lonsdale O (2017a) The *Liriomyza* (Diptera: Schizophora: Agromyzidae) of Canada and Alaska. Zootaxa 4234: 1–156. 10.11646/zootaxa.4234.1.1 28264361

[B148] Lonsdale O (2017b) World catalogue of the druid flies (Diptera: Schizophora: Clusiidae). Zootaxa 4333: 1–85. 10.11646/zootaxa.4333.1.1 29242451

[B149] Lonsdale O Cheung DKB Marshall SA (2011) Key to the World genera and North American species of Clusiidae (Diptera: Schizophora). Canadian Journal of Arthropod Identification 14. 10.3752/cjai.2010.14

[B150] Lonsdale O Marshall SA (2007) Revision of the New World *Heteromeringia* (Diptera: Clusiidae: Clusiodinae). Beiträge zur Entomologie 57(1): 37–80.

[B151] Lukashevich ED Ribeiro GC (2018) Mesozoic fossils and the phylogeny of Tipulomorpha (Insecta: Diptera). Journal of Systematic Palaeontology. 10.1080/14772019.2018.1448899

[B152] Malloch JR (1914) Notes on the dipterous genus *Chyromya* R-D. Proceedings of the Entomological Society of Washington 16: 179–181.

[B153] Malloch JR (1933) Acalyptrata. Diptera of Patagonia and South Chile 6: 177–391.

[B154] Malloch JR McAtee WL (1924) Keys to flies of the families Lonchaeidae, Pallopteridae, and Sapromyzidae of the eastern United States, with a list of the species of the District of Columbia region. Proceedings of the United States National Museum 65(12): 1–26. 10.5479/si.00963801.65-2525.1

[B155] Marinho MAT Junqueira ACM Paulo DF Esposito MC Villet MH Azeredo-Espin AML (2012) Molecular phylogenetics of Oestroidea (Diptera: Calyptratae) with emphasis on Calliphoridae: insights into the interfamilial relationships and additional evidence for paraphyly among blowflies. Molecular Phylogenetics and Evolution 65: 840–854. 10.1016/j.ympev.2012.08.007 22926310

[B156] Marshall SA (1982) A revision of the Nearctic *Leptocera* (*Thoracochaeta* Duda) (Diptera: Sphaeroceridae). The Canadian Entomologist 114: 63–78. 10.4039/Ent11463-1

[B157] Marshall SA (2012) Flies: The Natural History and Diversity of Diptera. Firefly Books, Richmond Hill, Ontario, 616 pp.

[B158] Marshall SA Buck M (2010) Sphaeroceridae (Small Dung Flies). In: Brown BV, Borkent A, Cumming JM, Wood DM, Woodley NE, Zumbado MA (Coordinators), Manual of Central American Diptera, Volume 2. NRC Research Press, Ottawa, 1165–1187.

[B159] Marshall SA Richards OW (1987) Sphaeroceridae In: McAlpine JF, Peterson BV, Shewell GE, Teskey HJ, Vockeroth JR, Wood DM (Coordinators) Manual of Nearctic Diptera, Volume 2. Agriculture Canada Monograph 28, 993–1006.

[B160] Marshall SA Whitworth TL Roscoe L (2011) Blow flies (Diptera: Calliphoridae) of eastern Canada with a key to Calliphoridae subfamilies and genera of eastern North America, and a key to eastern Canadian species of Calliphorinae, Luciliinae, and Chrysomyinae. Canadian Journal of Arthropod Identification 11: 1–93. 10.3752/cjai.2011.11

[B161] Marshall SA Woodley NE Hauser M (2015) The historical spread of the Black Soldier Fly, *Hermetiaillucens* (L.) (Diptera, Stratiomyidae, Hermetiinae), and its establishment in Canada. Journal of the Entomological Society of Ontario 146: 51–54.

[B162] Mathis WN (2011a) World catalog and conspectus on the family Helcomyzidae (Diptera: Schizophora). Myia 12: 267–280.

[B163] Mathis WN (2011b) World catalog and conspectus on the family Heterocheilidae (Diptera: Schizophora). Myia 12: 281–289.

[B164] Mathis W Barraclough D (2011) World catalog and conspectus on the Family Diastatidae (Diptera: Schizophora). Myia 12: 235–266.

[B165] Mathis WN McAlpine DK (2011) A catalog and conspectus on the Family Coelopidae (Diptera: Schizophora). Myia 12: 171–205.

[B166] Mathis WN Rung A (2011) World catalog and conspectus on the Family Periscelididae (Diptera: Schizophora). Myia 12: 341–377

[B167] Mathis WN Sueyoshi M (2011) World catalog and conspectus on the family Dryomyzidae (Diptera: Schizophora). Myia 12: 207–233.

[B168] Mathis WN Zatwarnicki T (1995) World catalog of Shore Flies (Diptera: Ephydridae). Associated Publishers, Gainesville, 423 pp.

[B169] Mattingly PF (1971) Illustrated keys to the genera of mosquitoes. Contributions of the American Entomological Institute 7(4): 1–84.

[B170] McAlpine DK (1973) The Australian Platystomatidae (Diptera, Schizophora) with a revision of five genera. Australian Museum Memoir 15: 1–256. 10.3853/j.0067-1967.15.1973.454

[B171] McAlpine DK (1975) The subfamily classification of the Micropezidae and the genera of Eurybatinae (Diptera: Schizophora). Journal of Entomology (B) 43(2): 231–245. 10.1111/j.1365-3113.1975.tb00133.x

[B172] McAlpine DK (1985) The Australian genera of Heleomyzidae (Diptera: Schizophora) and a reclassification of the family into tribes. Records of the Australian Museum 36: 203–251. 10.3853/j.0067-1975.36.1985.346

[B173] McAlpine DK (1991a) Review of the Australian kelp flies (Diptera: Coelopidae). Systematic Entomology 16: 29–84. 10.1111/j.1365-3113.1991.tb00573.x

[B174] McAlpine DK (1991b) Relationships of the genus *Heterocheila* (Diptera: Sciomyzoidea) with description of a new family. Tijdschrift voor Entomlogie 134: 193–199.

[B175] McAlpine DK (1997a) Relationships of the Megamerinidae. Beiträge zur Entomologie 47(2): 465–475.

[B176] McAlpine DK (1997b) Gobryidae, a new family of acalyptrate flies (Diptera: Diopsoidea), and a discussion of relationships of the diopsoid families. Records of the Australian Museum 49: 167–194. 10.3853/j.0067-1975.49.1997.1264

[B177] McAlpine DK (1998) Family Cypselosomatidae. In: Papp L Darvas B (Eds) Contributions to a Manual of Palaearctic Diptera, Volume 3.Science Herald, Budapest, 151–154.

[B178] McAlpine DK (2007a) The Surge Flies (Diptera: Canacidae: Zaleinae) of Australasia and notes on Tethinid-Canacid morphology and relationships. Records of the Australian Museum 59: 27–64. 10.3853/j.0067-1975.59.2007.1468

[B179] McAlpine DK (2007b) Review of the Borboroidini or Wombat Flies (Diptera: Heteromyzidae), with reconsideration of the status of families Heleomyzidae and Sphaeroceridae, and descriptions of femoral gland-baskets. Records of the Australian Museum 59(3): 143–219. 10.3853/j.0067-1975.59.2007.1487

[B180] McAlpine JF (1977) A revised classification of the Piophilidae including ‘Neottiophilidae’ and ‘Thyreophoridae’ (Diptera: Schizophora). Memoirs of the Entomological Society of Canada 103: 1–66. 10.4039/entm109103fv

[B181] McAlpine JF (1987) Lonchaeidae In: McAlpine JF, Peterson BV, Shewell GE, Teskey HJ, Vockeroth JR, Wood DM (Coordinators) Manual of Nearctic Diptera, Volume 2. Agriculture Canada Monograph 28, 791–797.

[B182] McAlpine JF (1989) Phylogeny and classification of the Muscomorpha In: McAlpine JF, Wood DM (Coordinators) Manual of Nearctic Diptera, Volume 3. Agriculture Canada Monograph 32, 1397–1518.

[B183] McAlpine JF Downes JA Oliver DR Peterson BV Shewell GE Teskey HJ Vockeroth JR Wood DM (1979) Diptera. In: Danks HV (Ed.) Canada and its insect fauna.Memoirs of the Entomological Society of Canada No. 108, 389–424. 10.4039/entm111108389-1

[B184] McAlpine JF Peterson BV Shewell GE Teskey HJ Vockeroth JR Wood DM [Coordinators] (1981) Manual of Nearctic Diptera, Volume 1. Agriculture Canada Monograph 27, 1–674.

[B185] McAlpine JF Peterson BV Shewell GE Teskey HJ Vockeroth JR Wood DM [Coordinators] (1987) Manual of Nearctic Diptera, Volume 2. Agriculture Canada Monograph 28: 675–1332.

[B186] McAlpine JF Wood DM [Coordinators] (1989) Manual of Nearctic Diptera, Volume 3. Agriculture Canada Monograph 32, 1333–1581.

[B187] McKeever S French FE (1991) *Corethrella* (Diptera: Corethrellidae) of North America north of Mexico: distribution and morphology of immature stages. Annals of the Entomological Society of America 84: 522–530. 10.1093/aesa/84.5.522

[B188] Melander AL (1920) Synopsis of the dipterous family Psilidae. Psyche 27: 91–101. 10.1155/1920/42460

[B189] Merritt RW Peterson BV (1976) A synopsis of the Micropezidae (Diptera) of Canada and Alaska, with description of four new species. Canadian Journal of Zoology 54(9): 1488–1506. 10.1139/z76-172

[B190] Michelsen V (1996) Neodiptera: new insights into the adult morphology and higher level phylogeny of Diptera (Insecta). Zoological Journal of the Linnean Society 117: 71–102. 10.1111/j.1096-3642.1996.tb02149.x

[B191] Miller BR Crabtree MB Savage HM (1997) Phylogenetic relationships of the Culicomorpha inferred from 18S and 5.8S ribosomal DNA sequences (Diptera: Nematocera). Insect Molecular Biology 6: 105–114. 10.1111/j.1365-2583.1997.tb00078.x 9099574

[B192] Miller ME Marshall SA Grimaldi DA (2017) A review of the species of *Drosophila* (Diptera: Drosophilidae) and genera of Drosophilidae of northeastern North America. Canadian Journal of Arthropod Identification 31: 1–282.

[B193] Mohrig W Heller K Hippa H Vilkamaa P Menzel F (2013) Revision of the black fungus gnats (Diptera: Sciaridae) of North America. Studia Dipterologica 19(2012): 141–286.

[B194] Moores A Savage J (2005) A taxonomic revision of *Piezura* Rondani (Diptera: Fanniidae). Zootaxa 1096: 41–59. 10.11646/zootaxa.1096.1.4

[B195] Moulton JK (2017) The true identity of *Dixamodesta* Johannsen (Diptera: Dixidae) resolved: synonymy of *Dixasimilis* Johannsen, designation of the *Dixaubiquita* species group, and description of three new eastern Nearctic species. Zootaxa 4216: 247–260. 10.11646/zootaxa.4216.3.3 28183120

[B196] Moulton JK Wiegmann BM (2004) Evolution and phylogenetic utility of CAD (rudimentary) among Mesozoic-aged Eremoneuran Diptera (Insecta). Molecular Phylogenetics and Evolution 31: 363–378. 10.1016/S1055-7903(03)00284-7 15019631

[B197] Munari L Mathis WN (2010) World Catalog of the Family Canacidae (including Tethinidae) (Diptera), with keys to the supraspecific taxa. Zootaxa 2471: 1–84.

[B198] Munroe DD (1974) The systematics, phylogeny, and zoogeography of *Symmerus* Walker and *Australosymmerus* Freeman (Diptera: Mycetophilidae: Ditomyiinae). Memoirs of the Entomological Society of Canada 92: 1–183. 10.4039/entm10692fv

[B199] Namba R (1956) A revision of the flies of the genus *Rivellia* (Ortalidae, Diptera). Proceedings of the United Stated National Museum 106: 21–84. 10.5479/si.00963801.106-3363.21

[B200] Nartshuk EP (2012) A check list of the world genera of the family Chloropidae (Diptera, Cyclorrhapha, Muscomorpha). Zootaxa 3267: 1–43.

[B201] Nirmala X Hypša V Žurovec M (2001) Molecular phylogeny of Calyptratae (Diptera: Brachycera): the evolution of 18S and 16S ribosomal rDNAs in higher dipterans and their use in phylogenetic inference. Insect Molecular Biology 10(5): 475–485. 10.1046/j.0962-1075.2001.00286.x 11881812

[B202] Norrbom AL Korytkowski CA (2010) Lonchaeidae (lance flies). In: Brown BV Borkent A Cumming JM Wood DM Woodley NE Zumbado MA (Eds) Manual of Central American Diptera, Volume 2.National Research Council Press, Ottawa, 857–863.

[B203] O’Hara JE (1983) Classification, phylogeny and zoogeography of the North American species of *Siphona* Meigen (Diptera: Tachinidae). Quaestiones Entomologicae 18 [1982]: 261–380.

[B204] O’Hara JE (1989) Systematics of the genus group taxa of the Siphonini (Diptera: Tachinidae). Quaestiones Entomologicae 25: 1–229.

[B205] O’Hara JE (1994) Revision of Nearctic species of *Ceromya* Robineau-Desvoidy (Diptera: Tachinidae). The Canadian Entomologist 126: 775–806. 10.4039/Ent126775-3

[B206] O’Hara JE (2002) Revision of the Polideini (Tachinidae) of America north of Mexico. Studia Dipterologica. Supplement 10, 170 pp.

[B207] O’Hara JE (2012) Review of *Euthera* (Diptera: Tachinidae) in North America with the description of a new species. The Canadian Entomologist 144: 206–215. 10.4039/tce.2012.23

[B208] O’Hara JE Cerretti P Dahlem GA (2015) First North American record of the Palaearctic rhinophorid *Steveniadeceptoria* (Loew) (Diptera: Rhinophoridae). Zootaxa 4058(2): 293–295. 10.11646/zootaxa.4058.2.11 26701527

[B209] O’Hara JE Wood DM (2004) Catalogue of the Tachinidae (Diptera) of America north of Mexico. Memoirs on Entomology, International 18, iv + 410 pp.

[B210] Oosterbroek P (1984) A revision of the crane-fly genus *Nephrotoma* Meigen, 1803, in North America (Diptera, Tipulidae), Part II, the non-*dorsalis* species-groups. Beaufortia 34: 117–180.

[B211] Oosterbroek P (2018) Catalogue of the craneflies of the world (Diptera, Tipuloidea: Pediciidae, Limoniidae, Cylindrotomidae, Tipulidae) [online]. http://ccw.naturalis.nl [accessed 19.III.2018].

[B212] Oosterbroek P Courtney G (1995) Phylogeny of the nematocerous families of Diptera (Insecta). Zoological Journal of the Linnean Society 115: 267–311. 10.1111/j.1096-3642.1995.tb02462.x

[B213] Ozerov AL (2005) World catalogue of the family Sepsidae (Insecta: Diptera). Zoologicheskiĭ muzeĭ, Moscow, 74 pp.

[B214] Pape T (1992) Phylogeny of the Tachinidae family-group (Diptera: Calyptratae). Tijdschrift voor Entomologie 135: 43–86.

[B215] Pape T (1996) Catalogue of the Sarcophagidae of the world (Insecta: Diptera). Memoirs on Entomology, International 8: 1–557.

[B216] Pape T Blagoderov V Mostovski MB (2011) Order DIPTERA Linnaeus, 1758. In: Zhang Z-Q (Ed.) Animal biodiversity: An outline of higher-level classification and survey of taxonomic richness.Zootaxa 3148: 222–229. 10.11646/zootaxa.3703.1.1 26146682

[B217] Pape T Thompson FC (Eds) (2013) Systema Dipterorum, Version 1.5 www.diptera.dk [accessed 18.VI.2018]

[B218] Papp L (1998) Families of Heleomyzoidea. In: Papp L Darvas B (Eds) Contributions to a Manual of Palaearctic Diptera.Volume 3. Higher Brachycera. Science Herald, Budapest, 425–455.

[B219] Papp L (2010) A study on Hesperinus Walker with description of a new species (Diptera: Hesperinidae). Acta Zoologica Academiae Scientiarum Hungaricae 56(4): 347–370.

[B220] Pauli T Burt TO Meusemann K Bayless K Donath A Podsiadlowski L Mayer C Kozlof A Vasilikipoulos A Liu S Zhou X Yeates D Misof B Peters RS Mengual X (2018) New data, same story: phylogenomics does not support Syrphoidea (Diptera: Syrphidae, Pipunculidae). Systematic Entomology 43(3): 447–459. 10.1111/syen.12283

[B221] Pawlowski J Szadziewski R Kmieciak D Fahrni J Bittar G (1996) Phylogeny of the infraorder Culicomorpha (Diptera: Nematocera) based on 28S RNA gene sequences. Systematic Entomology 21: 167–178. 10.1046/j.1365-3113.1996.d01-5.x

[B222] Pivar RJ Moulton JK Sinclair BJ (2018) Revision of the Western Nearctic *Androprosopa* (Diptera: Thaumaleidae) and descriptions of three new species. Insect Systematics & Evolution. 10.1163/1876312X-00002189

[B223] Pollet MAA Brooks SE Cumming JM (2004) Catalog of the Dolichopodidae (Diptera) of America north of Mexico. Bulletin of the American Museum of Natural History 283: 1–114. 10.1206/0003-0090(2004)283<0001:COTDDO>2.0.CO;2

[B224] Pont AC (1984) A revision of the Fanniidae and Muscidae (Diptera) described by Fallén. Entomologica Scandinavica 15(2): 277–297. 10.1163/187631284X00235

[B225] Pont AC (2011) The Muscidae described by J.W. Zetterstedt (Insecta: Diptera). Zootaxa 2852: 1–83.

[B226] Pratt HD (1992) Key to the winter crane flies of North America (Diptera: Trichoceridae). Acta Zoologica Cracoviensia 35(1): 79–85.

[B227] Pratt GK Pratt HD (1980) Notes of Nearctic *Sylvicola* (Diptera: Anisopodidae). Proceedings of the Entomological Society of Washington 82(1): 86–98.

[B228] Quate LW (1955) A revision of the Psychodidae (Diptera) in America north of Mexico. University of California Publications in Entomology 10: 103–273.

[B229] Rader R Bartomeus I Garibaldi LA Garratt MPD Howlett BG Winfree R Cunningham SA Mayfield MM Arthur AD Andersson GKS Bommarco R Brittain C Carvalheiro LG Chacoff NP Entling MH Foully B Freitas BM Gemmill-Herren B Ghazoul J Griffin SR Gross CL Herbertsson L Herzog F Hipólito J Jaggar S Jauker F Klein A-M Kleijn D Krishnan S Lemos CQ Lindström SAM Mandelik Y Monteiro VM Nelson W Nilsson L Pattemore DE Pereira NO Pisanty G Potts SG Reemer M Rundlöf M Sheffield CS Scheper J Schüepp C Smith HG Stanley DA Stout JC Szentgyörgyi H Taki H Vergara CH Viana BF Woyciechowski M (2016) Non-bee insects are important contributors to global crop pollination. Proceedings of the National Academy of Sciences 113: 146–151. 10.1073/pnas.1517092112 PMC4711867 26621730

[B230] Ratnasingham S Hebert PD (2013) A DNA-based registry for all animal species: The barcode Index Number (BIN) system. PLOS One 8(7) e66213. 10.1371/journal.pone.0066213 PMC3704603 23861743

[B231] Remsen J O’Grady P (2002) Phylogeny of Drosophilinae (Diptera: Drosophilidae), with comments on combined analysis and character support. Molecular Phylogenetics and Evolution 24: 249–264. 10.1016/S1055-7903(02)00226-9 12144760

[B232] Renaud AK Savage J Adamowicz SJ (2012b) DNA barcoding of Northern Nearctic Muscidae (Diptera) reveals high correspondence between morphological and molecular species limits. BMC Ecology 12: 24. 10.1186/1472-6785-12-24 PMC3537539 23173946

[B233] Renaud AK Savage J Roughley RE (2012a) Muscidae (Diptera) diversity in Churchill, Canada, between two time periods: evidence for limited changes since the Canadian Northern Insect Survey. The Canadian Entomologist 144: 29–51. 10.4039/tce.2012.6

[B234] Rivera J Currie DC (2009) Identification of Nearctic black flies using DNA barcodes (Diptera: Simuliidae). Molecular Ecology Resources suppl s 1: 224–236. 10.1111/j.1755-0998.2009.02648.x. 21564982

[B235] Roback SS (1951) A Classification of the Muscoid Calyptrate Diptera. Annals of the Entomological Society of America 44: 327–361. 10.1093/aesa/44.3.327

[B236] Rochefort S Wheeler TA (2015) Diversity of Piophilidae (Diptera) in northern Canada and description of a new Holarctic species of *Parapiophila* McAlpine. Zootaxa 3925: 229–240. 10.11646/zootaxa.3925.2.5 25781741

[B237] Rognes K (1991) Blowflies (Diptera, Calliphoridae) of Fennoscandia and Denmark. Fauna Entomologica Scandinavica 24: 1–272.

[B238] Roháček J Barber K (2016) Nearctic Anthomyzidae: a monograph of *Anthomyza* and allied genera (Diptera). Acta Entomologica Musei Nationalis Pragae 56 (suppl.): 1–412.

[B239] Roháček J Marshall SA Norrbom AL Buck M Quiros DI Smith I (2001) World catalog of Sphaeroceridae (Diptera). Slezské zemské museum, Opava, 414 pp.

[B240] Roth D Henry B Mak S Fraser M Taylor M Li M Cooper K Furnell A Wong Q Morshed M (2010) West Nile Virus range expansion into British Columbia. Emerging Infectious Diseases 16(8): 1251–1258. 10.3201/eid1608.100483 20678319 PMC3298306

[B241] Rotheray GE Horsfield D (2013) Development sites and early stages of eleven species of Clusiidae (Diptera) occurring in Europe. Zootaxa 3619: 401–427. 10.11646/zootaxa.3619.4.1 26131483

[B242] Rung A Mathis WN (2011) A Revision of the Genus *Aulacigaster* Macquart (Diptera: Aulacigastridae). Smithsonian Contributions to Zoology 633: xii + 1–132. 10.5479/si.00810282.633

[B243] Sabrosky CW Bennett GF Whitworth TL (1989) Bird blowflies (*Protocalliphora*) in North America (Diptera: Calliphoridae), with notes on Palearctic species. Smithsonian Institute Press, Washington, DC, 312 pp 10.5962/bhl.title.46311

[B244] Sæther OA (1972) VI. Chaoboridae. In: Zooplankton der Binnengewässer. 1. Tiel. Binnengewässer26: 257–280.

[B245] Sæther OA (2000) Phylogeny of Culicomorpha (Diptera). Systematic Entomology 25: 223–234.

[B246] Savage J (2003) Revision of the genus *Thricops* Rondani (Diptera: Muscidae). Entomologica Scandinavica Supplement 61: 1–143

[B247] Schlinger EI Gillung JP Borkent CJ (2013) New spider flies from the Neotropical Region (Diptera, Acroceridae) with a key to New World genera. ZooKeys 270: 59–93. 10.3897/zookeys.270.4476 PMC3668421 23730188

[B248] Schneeberg K Courtney GW Beutel RG (2011) Adult head structures of Deuterophlebiidae (Insecta), a highly derived “ancestral” dipteran lineage. Arthropod Structure & Development 40: 93–104. 10.1016/j.asd.2010.07.002 20637300

[B249] Schneeberg K Friedrich F Courtney GW Wipfler B Beutel RG (2012) The larvae of Nymphomyiidae (Diptera, Insecta) - ancestral and highly derived? Arthropod Structure & Development 41: 293–301. 10.1016/j.asd.2012.01.002 22349310

[B250] Sellers RF Maarouf AR (1991) Possible introduction of epizootic hemorrhagic disease of deer virus (serotype 2) and bluetongue virus (serotype 11) into British Columbia in 1987 and 1988 by infected *Culicoides* carried on the wind. Canadian Journal of Veterinary Research 55: 367–370. 1665099 PMC1263485

[B251] Ševčík J Kaspřák D Mantič M Fitzgerald S Ševčíková T Tóthová A Jaschhof M (2016) Molecular phylogeny of the megadiverse insect infraorder Bibionomorpha sensu lato (Diptera). PeerJ 4: e2563. 10.7717/peerj.2563 PMC5075709 27781163

[B252] Shin S Bayless KM Winterton SL Dikow T Lessard BD Yeates DK Wiegmann BM Trautwein MD (2017) Taxon sampling to address an ancient rapid radiation: a supermatrix phylogeny of early brachyceran flies (Diptera). Systematic Entomology 43: 277–289. 10.1111/syen.12275

[B253] Sinclair BJ (1988) The madicolous Tipulidae (Diptera) of eastern North America, with descriptions of the biology and immature stages of *Dactylolabismontana* (Osten Sacken) and *D.hudsonica* Alexander (Diptera: Tipulidae). The Canadian Entomologist 120: 569–573. 10.4039/Ent120569-6

[B254] Sinclair B.J. (1996) A review of the Thaumaleidae (Diptera: Culicomorpha) of eastern North America, including a redefinition of the genus *Androprosopa* Mik. Entomologica Scandinavica 27: 361–376. 10.1163/187631296X00124

[B255] Sinclair BJ (2013) Rediscovered at last: a new enigmatic genus of Axymyiidae (Diptera) from western North America. Zootaxa 3682: 143–150. 10.11646/zootaxa.3682.1.7 25243280

[B256] Sinclair BJ Borkent A Wood DM (2007) The male genital tract and aedeagal components of the Diptera with a discussion of their phylogenetic significance. Zoological Journal of the Linnean Society 150: 711–742. https://doi.org/10.1111/j.1096-3642.2007.00314.x

[B257] Sinclair BJ Brooks SE Cumming JM Coovert GA (2011) Revision of the Nearctic species of *Heleodromia* (Diptera: Empidoidea: Brachystomatidae). The Canadian Entomologist 143: 629–651. 10.4039/n11-036

[B258] Sinclair BJ Cumming JM (2006) The morphology, higher-level phylogeny and classification of the Empidoidea (Diptera). Zootaxa 1180: 1–172.

[B259] Sinclair BJ Cumming JM (2017) 52. Hybotidae (hybotid dance flies). In: Kirk-Spriggs AH Sinclair BJ (Eds) Manual of Afrotropical Diptera.Volume 2. Nematocerous Diptera and lower Brachycera. Suricata 5, South African National Biodiversity Institute, Pretoria, 1237–1250.

[B260] Sinclair BJ Cumming JM Brooks SE (2013) Male terminalia of Diptera (Insecta): a review of evolutionary trends, homology and phylogenetic implications. Insects Systematics & Evolution 44: 373–415. 10.1163/1876312X-04401001

[B261] Sinclair BJ Cumming JM Wood DM (1994) Homology and phylogenetic implications of male genitalia in Diptera – Lower Brachycera. Entomologica Scandinavica 24: 407–432. 10.1163/187631293X00190

[B262] Sinclair BJ MacDonald JF (2012) Revision of *Dolichocephala* of America, north of Mexico (Diptera: Empididae: Clinocerinae). The Canadian Entomologist 144: 62–80. 10.4039/tce.2012.8

[B263] Skevington J Marshall SA (1997) First record of a big-headed fly, *Eudorylasalternatus* (Cresson) (Diptera: Pipunculidae), reared from the subfamily Cicadellinae (Homoptera: Cicadellidae), with an overview of pipunculid-host associations in the Nearctic Region. The Canadian Entomologist 129: 387–398. 10.4039/Ent129387-3

[B264] Spencer KA (1969) The Agromyzidae of Canada and Alaska. Memoirs of the Entomological Society of Canada 64: 1–311. 10.4039/entm10164fv

[B265] Starý J (1992) Phylogeny and classification of Tipulomorpha, with special emphasis on the family Limoniidae. Acta Zoologica Cracoviensia 35: 11–36.

[B266] Starý J Brodo F (2009) Arctic species of the subgenus Symplecta sensu stricto (Diptera: Limoniidae). The Canadian Entomologist 141: 1–30. 10.4039/n08-031

[B267] Steyskal GC (1978) Synopsis of the North American Pyrgotidae (Diptera). Proceedings of the Entomological Society of Washington 80: 149–155.

[B268] Steyskal GC (1987a) Otitidae In: McAlpine JF, Peterson BV, Shewell GE, Teskey HJ, Vockeroth JR, Wood DM (Coordinators) Manual of Nearctic Diptera, Volume 2. Agriculture Canada Monograph 28, 799–80.

[B269] Steyskal GC (1987b) Platystomatidae In: McAlpine JF, Peterson BV, Shewell GE, Teskey HJ, Vockeroth JR, Wood DM (Coordinators) Manual of Nearctic Diptera, Volume 2. Agriculture Canada Monograph 28, 809–812.

[B270] Steyskal GC Knutson LV (1981) Empididae In: McAlpine JF, Peterson BV, Shewell GE, Teskey HJ, Vockeroth JR, Wood DM (Coordinators) Manual of Nearctic Diptera, Volume 1. Agriculture Canada Monograph 27, 607–624.

[B271] Stone A Sabrosky CW Wirth WW Foote RH Coulson JR (Eds) (1965) A catalog of the Diptera of America north of Mexico. United States Department of Agriculture, Agriculture Handbook No. 276, 1696 pp.

[B272] Stuke J-H (2016) Carnidae (Diptera) in the Canadian National Collection of Insects (Ottawa), with the description of five new species. Zootaxa 4084: 540–556. 10.11646/zootaxa.4084.4.5 27394280

[B273] Sun X-K Marshall SA (2003) Systematics of *Phasia* Latreille (Diptera: Tachinidae). Zootaxa 276: 1–320. 10.11646/zootaxa.276.1.1

[B274] Swann JE Kenner RD Cannings RA Copley CR (2006) *Exairetaspinigera* (Diptera: Stratiomyidae): the first published North American records of an Australian soldier fly. Journal of the Entomological Society of British Columbia 103: 71–72.

[B275] Tachi T (2014) Homology of the metapleuron of Cyclorrhapha, with discussion of the paraphyly of Syrphoidea (Diptera: Aschiza). Insect Systematics & Evolution 45(4): 395–414. 10.1163/1876312X-45012112

[B276] Tangelder IRM (1983) A revision of the Crane Fly genus *Nephrotoma* Meigen, 1803, in North America (Diptera, Tipulidae), Part 1, the *dorsalis* species-group. Beaufortia 33: 111–205.

[B277] Tantawi TI Whitworth TL Sinclair BJ (2017) Revision of the Nearctic *Calliphora* Robineau-Desvoidy (Diptera: Calliphoridae). Zootaxa 4226: 301–347. 10.11646/zootaxa.4226.3.1 28187619

[B278] Teskey HJ (1976) Diptera larvae associated with trees in North America. Memoirs of the Entomological Society of Canada 100: 1–83. 10.4039/entm108100fv

[B279] Teskey HJ (1990) The Insects and Arachnids of Canada. Part 16. The Horse Flies and Deer Flies of Canada and Alaska. Diptera: Tabanidae. Publication 1838, Research Branch, Agriculture Canada, Ottawa, 381 pp.

[B280] Thielman AC Hunter FF (2007) Photographic Key to the Adult Female Mosquitoes (Diptera: Culicidae) of Canada. Canadian Journal of Arthropod Identification 4: 1–16. 10.3752/cjai.2007.04

[B281] Thomas AW (2011) Tabanidae of Canada, east of the Rocky Mountains 2: a photographic key to the genera and species of Tabaninae (Diptera: Tabanidae). Canadian Journal of Arthropod Identification 13. 10.3752/cjai.2011.13

[B282] Thomas AW Marshall SA (2009) Tabanidae of Canada, east of the Rocky Mountains 1: a photographic key to the species of Chrysopsinae and Pangoniinae (Diptera: Tabanidae). Canadian Journal of Arthropod Identification 8. 10.3752/cjai.2009.08

[B283] Thompson FC (2009) Nearctic Diptera: twenty years later. In: Pape T Bickel D Meier R (Eds) Diptera Diversity: Status, Challenges and Tools.Brill, Leiden, Boston, 3–46. 10.1163/ej.9789004148970.I-459.6

[B284] Tkoč M Tóthová A Ståhls G Chandler PJ Vaňhara J (2017) Molecular phylogeny of flat-footed flies (Diptera: Platypezidae): main clades supported by new morphological evidence. Zoologica Scripta 46: 429–444. 10.1111/zsc.12222

[B285] Trautwein MD Wiegmann BM Yeates DK (2010) A multigene phylogeny of the fly superfamily Asiloidea (Insecta): Taxon sampling and additional genes reveal the sister-group to all higher flies (Cyclorrhapha). Molecular Phylogenetics and Evolution 56: 918–930. 10.1016/j.ympev.2010.04.017 20399874

[B286] Väisänen R (1984) A monograph of the genus *Mycomya* Rondani in the Holarctic region (Diptera, Mycetophilidae). Acta Zoologica Fennica 177: 1–346.

[B287] Valliant F (2002) Insecta: Diptera: Lonchopteridae. In: Schwoerbel J, Zwick P (Eds) Süßwasserfauna von Mitteleuropa. Volume 21, Insecta: Diptera, 22, 23, Lonchopteridae, Sciomyzidae. Spektrum Akademischer Verlag, Heidelberg and Berlin, 14 pp.

[B288] Vockeroth JR (1965) Family Coelopidae In: Stone A Sabrosky CW Wirth WW Foote RH Coulson JR (Eds) A catalog of the Diptera of America north of Mexico.United States Department of Agriculture, Agriculture Handbook No. 276, 679–680.

[B289] Vockeroth JR (1981) Mycetophilidae In: McAlpine JF, Peterson BV, Shewell GE, Teskey HJ, Vockeroth JR, Wood DM (Coordinators) Manual of Nearctic Diptera. Volume 1. Agriculture Canada Monograph 27, 223–246.

[B290] Wahlberg E Johanson KA (2018) Molecular phylogenetics reveals novel relationships within Empidoidea (Diptera). Systematic Entomology 43: 610–636. 10.1111/syen.12297

[B291] Webb DW (1974) A revision of the genus *Hilarimorpha* (Diptera: Hilarimorphidae). Journal of the Kansas Entomological Society 47: 172–222.

[B292] Webb DW (1975) New species of *Hilarimorpha* (Diptera: Hilarimorphidae). Entomological News 86: 80–84.

[B293] Webb DW (1984) A revision of the Nearctic species of the family Solvidae (Insecta: Diptera). Transactions of the American Entomological Society 110(3): 245–293.

[B294] Webb DW Gaimari SD Hauser M Holston KC Metz MA Irwin ME Kampmeier GE Algmin K (2013) An annotated catalogue of the New World Therevidae (Insecta: Diptera: Asiloidea). Zootaxa 3600: 1–105. 10.11646/zootaxa.3600.1.1 24614059

[B295] Wenzel RL (1965) Family Nycteribiidae In: Stone A, Sabrosky CW, Wirth WW, Foote RH, Coulson JR (Eds) A catalog of the Diptera of America north of Mexico. United States Department of Agriculture, Agriculture Handbook No. 276, 922.

[B296] Wheeler MR (1961) New species of southwestern Acalyptrate Diptera. The Southwestern Naturalist 6(2): 86–91. 10.2307/3669590

[B297] Wheeler TA Vockeroth JR Boucher S (1999) *Geomyzatripunctata* Fallén, a Palearctic opomyzid fly new to North America, with notes on range expansions into Holarctic Opomyzidae (Diptera). Journal of the Entomological Society of Ontario 130: 15–20.

[B298] Whitworth T (2006) Keys to the genera and species of blow flies (Diptera: Calliphoridae) of America north of Mexico. Proceedings of the Entomological Society of Washington 108: 689–725.

[B299] Whitworth T Dawson RD Magalon H Beaydry E (2007) DNA barcoding cannot reliably identify species of the blowfly genus *Protocalliphora* (Diptera: Calliphoridae). Proceedings of the Royal Society B 274: 1731–1739. 10.1098/rspb.2007.0062 17472911 PMC2493573

[B300] Wiederholm T (1986) Chironomidae of the Holarctic region. Keys and diagnoses. Part 2. Pupae. Entomologica Scandinavica Supplement 28: 1–482.

[B301] Wiederholm T (1989) Chironomidae of the Holarctic region. Keys and diagnoses. Part 3. Adult males. Entomologica Scandinavica Supplement 34: 1–532.

[B302] Wiegmann BM Trautwein MD Winkler IS Barr NB Kim J-W Lambkin C Bertone MA Cassel BK Bayless KM Heimberg AM Wheeler BM Peterson KJ Pape T Sinclair BJ Skevington JH Blagoderov V Caravas J Kutty SN Schmidt-Ott U Kampmeier GE Thompson FC Grimaldi DA Beckenbach AT Courtney GW Friedrich M Meier R Yeates DK (2011) Episodic radiations in the fly tree of life. Proceedings of the National Academy of Sciences of the United States of America 108(14): 5690–5695. 10.1073/pnas.1012675108 21402926 PMC3078341

[B303] Wiegmann BM Tsaur S-C Webb DW Yeates DK Cassel BK (2000) Monophyly and relationships of the Tabanomorpha (Diptera: Brachycera) based on 28S ribosomal gene sequences. Annals of the Entomological Society of America 93(5): 1031–1038. 10.1603/0013-8746(2000)093[1031:MAROTT]2.0.CO;2

[B304] Wiegmann BM Yeates DK (2017) Phylogeny of the Diptera. In: Kirk-Spriggs AH Sinclair BJ (Eds) Manual of Afrotropical Diptera.Volume 1. Introductory chapters and keys to Diptera families. Suricata 4. South African National Biodiversity Institute, Pretoria, 253–265.

[B305] Wihlm MW Courtney GW (2011) The distribution and life history of *Axymyiafurcata* McAtee (Diptera: Axymyiidae), a wood inhabiting, semi-aquatic fly. Proceedings of the Entomological Society of Washington 113: 385–398. 10.4289/0013-8797.113.3.385

[B306] Wihlm MW Sam RB Courtney GW (2012) Morphology of *Axymyiafurcata* (Diptera: Axymyiidae), including scanning electron microscopy of all life stages. The Canadian Entomologist 144: 273–290. 10.4039/tce.2012.27

[B307] Winkler IS Rung A Scheffer SJ (2010) Hennig’s orphans revisited: Testing morphological hypotheses in the “Opomyzoidea” (Diptera: Schizophora). Molecular Phylogenetics and Evolution 54: 746–762. 10.1016/j.ympev.2009.12.016 20040375

[B308] Winkler IS Scheffer SJ Mitter C (2009) Molecular phylogeny and systematics of leaf-mining flies (Diptera: Agromyzidae): Delimitation of *Phytomyza* Fallén sensu lato and included species groups, with new insights on morphological and host-use evolution. Systematic Entomology 34: 260–292. 10.1111/j.1365-3113.2008.00462.x

[B309] Winterton SL Ware JL (2015) Phylogeny, divergence times and biogeography of window flies (Scenopinidae) and the therevoid clade (Diptera: Asiloidea) Systematic Entomology 40: 491–519. 10.1111/syen.12117

[B310] Winterton SL Wiegmann BM Schlinger EI (2007) Phylogeny and Bayesian divergence time estimations of small-headed flies (Diptera: Acroceridae) using multiple molecular markers. Molecular Phylogenetics and Evolution 43: 808–832. 10.1016/j.ympev.2006.08.015 17196837

[B311] Wipfler B Courtney GW Craig DA Beutel RG (2012) First *l*-CT-based 3D reconstruction of a Dipteran larva – the head morphology of *Protanyderus* (Tanyderidae) and its phylogenetic implications. Journal of Morphology 273: 968–980. 10.1002/jmor.20035 22592892

[B312] Wood DM (1981) Axymyiidae In: McAlpine JF, Peterson BV, Shewell GE, Teskey HJ, Vockeroth JR, Wood DM (Coordinators) Manual of Nearctic Diptera, Volume 1. Agriculture Canada Monograph 27, 209–212.

[B313] Wood DM (1985) A taxonomic conspectus of the Blondeliini of North and Central America and the West Indies (Diptera: Tachinidae). Memoirs of the Entomological Society of Canada 132: 1–130. 10.4039/entm117132fv

[B314] Wood DM Borkent A (1989) Phylogeny and classification of the Nematocera In: McAlpine JF, Wood DM (Coordinators) Manual of Nearctic Diptera, Volume 3. Agriculture Canada Monograph 32, 1333–1370.

[B315] Wood DM Dang PT Ellis RA (1979) The mosquitoes of Canada, Diptera: Culicidae. Insects and Arachnids of Canada Handbook Series, Part 6. Agriculture Canada, Ottawa, 390 pp.

[B316] Woodley NE (1989) Phylogeny and classification of the “Orthorrhaphous” Brachycera [Chapter] 115. In: McAlpine JF, Wood DM (Coordinators) Manual of Nearctic Diptera. Volume 3. Agriculture Canada Monograph 32, 1371–1395.

[B317] Woodley NE (2001) A world catalog of the Stratiomyidae (Insecta: Diptera). Myia 11: 1–475.

[B318] Woodley NE (2011a) A catalog of the world Stratiomyidae (Insecta: Diptera): a supplement with revisionary notes and errata. Myia 12: 379–415.

[B319] Woodley NE (2011b) A catalog of the world Xylomyidae (Insecta: Diptera). Myia 12: 417–453.

[B320] Woodley NE (2011c) A catalog of the world Xylophagidae (Insecta: Diptera). Myia 12: 455–500.

[B321] Woodley NE Borkent A Wheeler TA (2009) Phylogeny of the Diptera. In: Brown BV Borkent A Cumming JM Wood DM Woodley NE Zumbado MA (Eds) Manual of Central American Diptera.Volume 1. NRC Research Press, Ottawa, 79–94.

[B322] Yeates DK (1994) The cladistics and classification of the Bombyliidae (Diptera: Asiloidea). Bulletin of the American Museum of Natural History 219: 1–191.

[B323] Yeates DK (2002) Relationships of extant lower Brachycera (Diptera): a quantitative synthesis of morphological characters Zoologica Scripta 31: 105–121. https://doi.org/10.1046/j.0300-3256.2001.00077.x

[B324] Yeates DK Wiegmann BM (2012) The impact of the Manual of Nearctic Diptera on Phylogenetic Dipterology. The Canadian Entomologist 144: 197–205. 10.4039/tce.2012.22

[B325] Yeates DK Wiegmann BM Courtney GW Meier R Lambkin C Pape T (2007) Phylogeny and systematics of Diptera: two decades of progress and prospects. Zootaxa 1668: 565–590.

[B326] Young AD (2018) Systematics of lower Cyclorrhaphan Diptera, with an emphasis on Syrphidae. PhD Thesis, Carleton University, Ottawa, 300 pp.

[B327] Young CW (1987) A revision of the crane fly genus *Dicranoptycha* in North America. The University of Kansas Science Bulletin 53: 215–274.

[B328] Young DG Perkins PV (1984) Phlebotomine sand flies of North America (Diptera: Psychodidae). Journal of the American Mosquito Control Association 44: 263–304.

[B329] Zaitzev AI (1982) Fungus gnats of the genus *Sciophila* Meig. of the Holarctic. Akademia Nauk SSSR, Moscow, 76 pp. [In Russian]

[B330] Zatwarnicki T (1996) A new reconstruction of the origin of eremoneuran hypopygium and its implications for classification (Insecta: Diptera). Genus 7: 103–175.

[B331] Zhang D Yan L Zhang M Chu H Cao J Li K Hu D Pape T (2016) Phylogenetic inference of calyptrates, with the first mitogenomes for Gasterophilinae (Diptera: Oestridae) and Paramacronychiinae (Diptera: Sarcophagidae). International Journal of Biological Sciences 2(5): 489–504. 10.7150/ijbs.12148 PMC4807417 27019632

[B332] Zloty J Sinclair BJ Pritchard G (2005) Discovered in our backyards: a new genus and species of a new family from the Rocky Mountains of North America (Diptera, Tabanomorpha). Systematic Entomology 30: 248–266. 10.1111/j.1365-3113.2005.00270.x

